# Precision Radiation Oncology: Molecular Strategies to Enhance Anti-Tumour Responses to Radiotherapy—Beyond Direct Tumour Cell Killing

**DOI:** 10.3390/ijms27219396

**Published:** 2026-10-22

**Authors:** Vishit Patel, Asmita Raja, Madeha Khan, Alan A. Melcher, Irene Chong, Adam Young

**Affiliations:** 1 The Royal Marsden Hospital NHS Foundation Trust, London SW3 6JJ, UK; 2 The Institute of Cancer Research, London SW7 3RP, UK

**Keywords:** radiotherapy, DNA damage response, radiosensitisation, immunotherapy, cGAS–STING, tumour microenvironment, precision radiation oncology, radiogenomics, genomic-adjusted radiation dose (GARD), microbiome

## Abstract

Radiotherapy remains a cornerstone of cancer treatment, yet treatment response continues to be limited by tumour radioresistance and considerable biological heterogeneity. Although advances in radiation delivery have substantially improved dose conformity and reduced toxicity, increasing evidence suggests that optimising therapeutic outcomes will require greater integration of tumour biology into treatment strategies. This review examines emerging molecular approaches that seek to enhance anti-tumour responses to radiotherapy beyond direct tumour cell killing. We discuss the DNA damage response as a therapeutic target for radiosensitisation, highlighting the evolving role of DNA damage response inhibitors in promoting both tumour cytotoxicity and immune activation. We further examine the mechanisms linking radiation-induced DNA damage to anti-tumour immunity, including immunogenic cell death, cGAS–STING signalling, and remodelling of the tumour microenvironment, together with the opportunities and challenges of combining radiotherapy with immune checkpoint inhibition. Finally, we review emerging concepts in precision radiation oncology, including spatially tailored irradiation strategies, genomically informed radiation prescription, radiogenomics, and the influence of the gut microbiome on treatment response. Collectively, these advances support a shift from anatomically guided radiotherapy towards biologically personalised treatment, integrating molecular, immunological and genomic information with normal-tissue susceptibility to improve the therapeutic ratio and enable more precise, patient-centred cancer care.

## 1. Introduction

Radiotherapy (RT) remains one of the most widely utilised anti-cancer therapies, delivered to over half of all patients during the course of their disease and forming a cornerstone of both curative and palliative treatment strategies [1]. Technological advances over recent decades, including image guidance, intensity-modulated radiotherapy (IMRT), volumetric modulated arc therapy (VMAT), and stereotactic body radiotherapy (SBRT), have enabled increasingly conformal dose delivery, facilitating dose escalation whilst reducing normal tissue toxicity [2]. However, local recurrence within the irradiated field remains a significant clinical challenge across many tumour types, highlighting the persistent influence of tumour-intrinsic biology and mechanisms of radioresistance [3]. Yet radiotherapy prescription remains guided predominantly by anatomical and clinicopathological parameters, with comparatively limited integration of tumour biology into treatment decision-making.

Historically, the anti-tumour effects of ionising radiation have been attributed primarily to direct and indirect DNA damage, culminating in genomic instability and cancer cell death. The therapeutic efficacy of RT is enhanced by the intrinsic genomic vulnerabilities of malignant cells, many of which harbour defects in DNA damage response (DDR) pathways [4]. However, it is now clear that radiation exerts effects extending far beyond direct cytotoxicity. Radiation-induced DNA damage initiates a complex network of molecular and immunological responses involving innate immune sensing, cytokine signalling, and remodelling of the tumour microenvironment (TME), all of which may influence treatment outcome [5].

These observations have fundamentally altered our understanding of RT. Rather than functioning solely as a local cytotoxic therapy, RT is increasingly recognised as a potent modulator of anti-tumour immunity. Yet the immunological consequences of radiation remain highly context-dependent, capable of promoting both immune activation and immune suppression. This complexity likely contributes to the discrepancy between compelling preclinical data supporting RT–immunotherapy combinations and the more modest benefits observed in many clinical studies, highlighting the need for a deeper understanding of the molecular determinants of response [5].

As oncology increasingly embraces precision medicine, radiotherapy must similarly evolve beyond conventional population-based treatment paradigms. Precision radiation oncology can be defined as the integration of biological, clinical, imaging, and dosimetric information to optimise the therapeutic ratio for an individual patient. This encompasses several related but distinct objectives: predicting intrinsic tumour radiosensitivity and normal-tissue toxicity; selecting patients most likely to benefit from radiotherapy or a particular systemic combination; adapting radiation dose and fractionation; and personalising the spatial distribution of dose according to intratumour and normal-tissue heterogeneity. Precision radiotherapy therefore extends beyond increasingly accurate anatomical targeting to address whether, when, where, and how radiation should be delivered.

This review focuses on the molecular and immunological dimensions of precision radiation oncology rather than providing a disease-specific account of radiotherapy indications or delivery techniques. We examine how DNA damage response dependencies may be exploited for radiosensitisation, how radiation-induced damage engages innate and adaptive immunity, and how the tumour microenvironment influences treatment response. We then consider emerging spatially tailored irradiation strategies and the potential contributions of genomics, imaging-derived biomarkers, immune phenotype, and host factors to treatment individualisation. By integrating these previously fragmented areas within a translational framework, we aim to distinguish biological association from clinically actionable prediction and to identify the steps required to convert mechanistic insights into treatment selection, dose or fractionation adaptation, rational systemic combinations, and ultimately prospective clinical validation.

## 2. DNA Damage Response as a Foundation for Immunogenic Radiotherapy

### 2.1. DDR Pathways and Synthetic Lethality

To preserve genomic integrity, cells rely on a highly coordinated signalling network responsible for detecting, signalling, and repairing DNA damage. Collectively termed DDR, this system is activated following genotoxic insults and involves a complex interplay between sensor proteins, signal transducers, cell-cycle checkpoints, and repair machinery. Central to this process are checkpoint kinases including ataxia telangiectasia mutated (ATM), ATM and Rad3-related (ATR), checkpoint kinase 1 (CHK1), and DNA-dependent protein kinase (DNA-PK), which coordinate DNA repair, cell-cycle arrest, and cellular survival following genotoxic stress. The DDR plays a fundamental role in maintaining genomic stability, and its disruption is increasingly recognised as a defining feature of oncogenesis [6,7]. Indeed, defects in DDR pathways contribute to genomic instability, mutational accumulation, and malignant transformation, while simultaneously creating therapeutic vulnerabilities that may be exploited to enhance treatment response [8].

**Figure 1:**
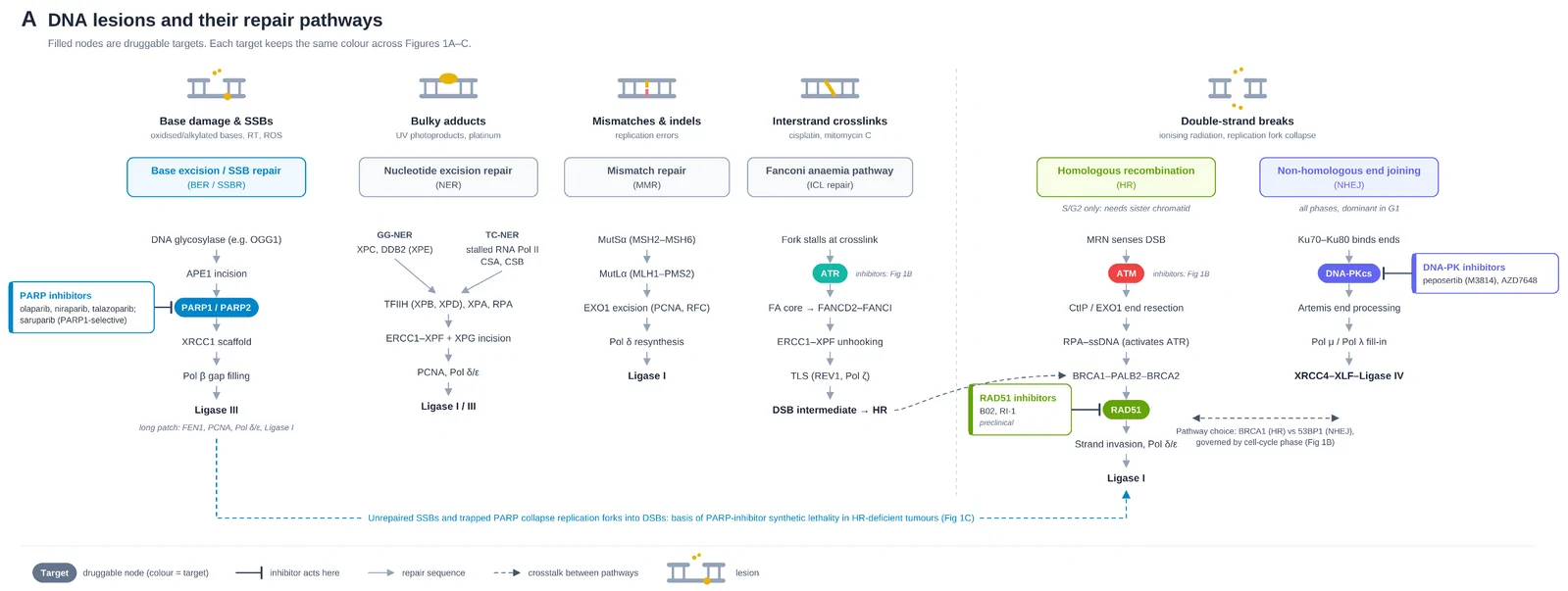

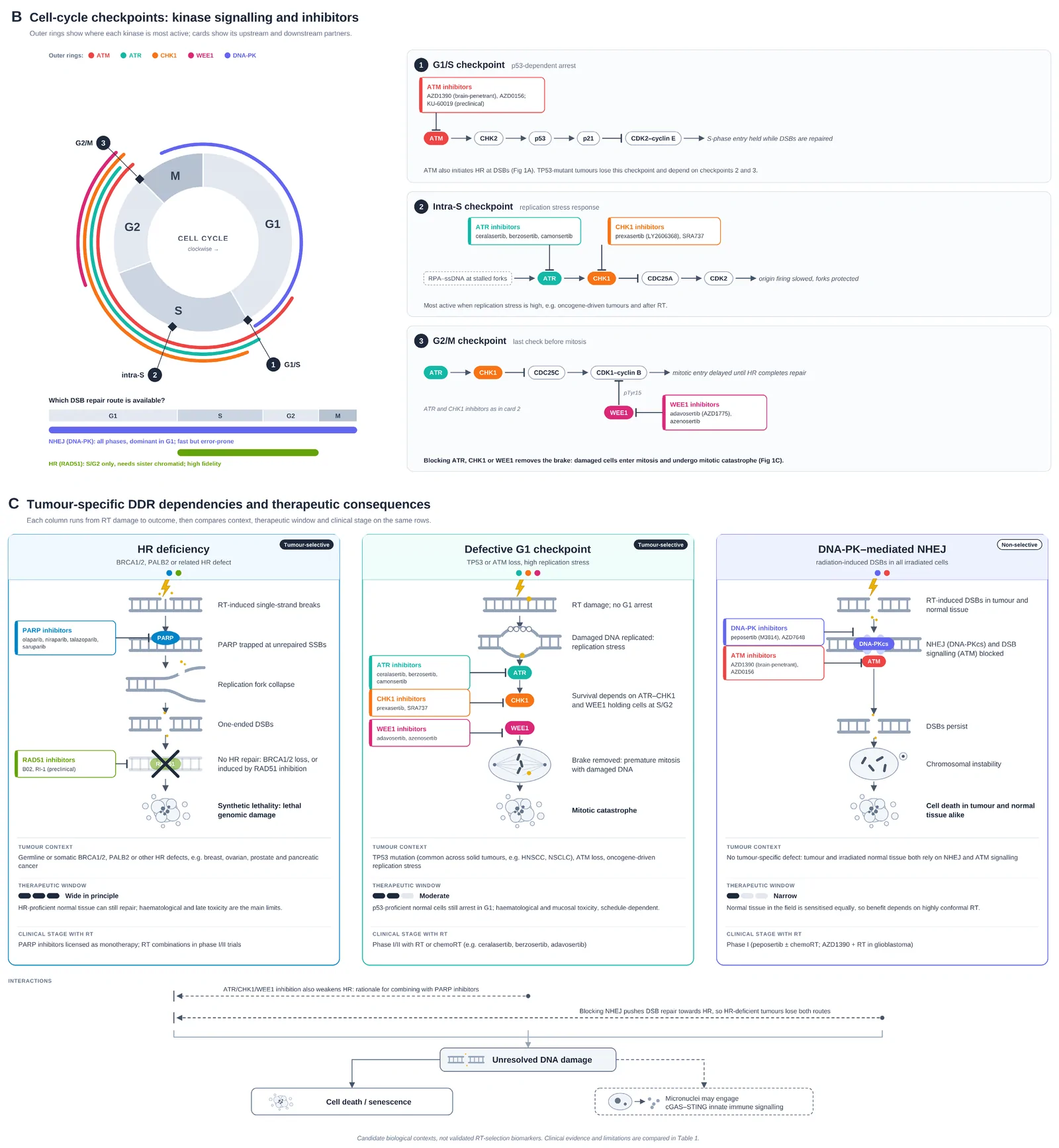
Radiation-induced DNA damage responses and candidate therapeutic dependencies. (**A**) Major forms of radiation-induced DNA damage and the principal pathways responsible for their repair, including base-excision and single-strand-break repair, nucleotide-excision repair, mismatch repair, the Fanconi anaemia pathway, homologous recombination and non-homologous end joining. The panel also illustrates repair-pathway crosstalk and the conversion of replication-associated lesions into double-strand breaks. (**B**) Cell-cycle checkpoint signalling following DNA damage and the principal sites of pharmacological inhibition within the ATM-CHK2, ATR-CHK1, WEE1 and DNA-PK pathways. (**C**) Candidate DNA-damage-response dependencies arising from homologous-recombination deficiency, defective G1-checkpoint control or reliance on DNA double-strand-break repair, together with their proposed therapeutic consequences and interactions. These biological contexts remain candidate rather than validated biomarkers for selecting radiotherapy combinations; the corresponding clinical evidence and translational limitations are summarised in Table 1.

As illustrated in Figure 1A, ionising radiation exerts its anti-tumour effects primarily through the induction of DNA damage, including base damage, single-strand breaks (SSBs) and double-strand breaks (DSBs), as well as clustered DNA lesions in which multiple lesions occur in close proximity. DSBs are considered particularly lethal because inadequate repair can result in chromosomal rearrangements, mitotic catastrophe or cell death. DSB repair occurs predominantly through two major pathways: homologous recombination (HR) and non-homologous end joining (NHEJ). HR facilitates high-fidelity repair through the use of a sister chromatid template and is therefore most active during the S and G2 phases of the cell cycle. In contrast, NHEJ functions throughout the cell cycle, particularly during G1, enabling rapid but more error-prone repair through direct ligation of DNA ends. Additional repair pathways, including base excision repair (BER), nucleotide excision repair (NER), and mismatch repair (MMR), contribute to the repair of oxidative damage, SSBs, and replication-associated errors [9,10].

The genomic instability characteristic of many tumours frequently results in dependence on residual DNA repair mechanisms, creating opportunities for therapeutic exploitation. These dependencies underpin the principle of synthetic lethality, whereby simultaneous disruption of complementary DNA repair mechanisms results in irreparable genomic instability and cell death. The clinical success of therapies targeting DNA repair vulnerabilities has validated DDR inhibition as an effective anti-cancer strategy and stimulated growing interest in combining DDR-targeted agents with radiotherapy to enhance tumour response [11]. The relationships between lesion-specific repair, cell-cycle checkpoint signalling and candidate tumour DDR dependencies are summarised in Figure 1A–C.

### 2.2. Radiation-Induced Cell Fates: Beyond Direct Cell Death

The biological consequences of radiation-induced DNA damage extend beyond a binary outcome of repair or cell death. Irradiated tumour cells may undergo apoptosis, necrotic forms of cell death, or loss of reproductive capacity following aberrant mitosis, while other cells enter prolonged cell-cycle arrest or senescence. Mitotic catastrophe describes an oncosuppressive process arising from premature or abnormal mitosis in the presence of unrepaired damage, which ultimately leads to cell death or irreversible loss of clonogenic potential rather than representing a distinct molecular death pathway. Radiation may also engage regulated processes including necroptosis and ferroptosis, while autophagy can either support cellular survival or contribute to treatment response depending on the biological context. The balance among these outcomes is influenced by radiation dose and fractionation, damage complexity, tumour lineage, oxygenation, cell-cycle state, TP53 status and the integrity of DNA repair and checkpoint pathways [4,12,13]. Importantly, cell fate may influence the subsequent immune response: preclinical evidence suggests, for example, that radiation-induced ZBP1-MLKL necroptotic signalling can promote cytosolic DNA accumulation, cGAS–STING activation and anti-tumour immunity [14].

These divergent responses provide a biological rationale for combining RT with inhibitors of compensatory DDR pathways. Tumours with defective G1 checkpoint control may become particularly reliant on ATR-CHK1- and WEE1-mediated S- and G2/M-checkpoint signalling; inhibition of these pathways can force cells containing unrepaired DNA into mitosis, thereby promoting mitotic catastrophe. Similarly, PARP inhibition increases replication-associated DNA damage, whereas inhibition of ATM or DNA-PK impairs the coordination or repair of radiation-induced DSBs. Although these combinations can increase tumour cell killing, radiosensitisation should not be considered synonymous with synthetic lethality. Classical synthetic lethality describes a setting in which concurrent disruption of two complementary pathways is lethal despite either defect being individually tolerated, whereas radiosensitisation encompasses any intervention that increases the biological effect of radiation [9,15,16].

One particularly important radiation-induced cell fate is cellular senescence: a state of sustained proliferative arrest commonly associated with persistent DDR signalling and activation of the p53-p21 and/or p16-RB pathways. Senescent cells remain metabolically active and may develop a senescence-associated secretory phenotype (SASP), characterised by the release of cytokines, chemokines, growth factors and proteases. The consequences are context-dependent. Senescence can suppress tumour growth and promote immune-mediated clearance; in an experimental model of radiation-induced osteosarcoma, RB1-dependent senescence and interleukin-6 (IL-6) signalling supported natural killer T-cell recruitment and tumour immune surveillance [17]. Conversely, persistent senescent tumour or stromal cells may promote chronic inflammation, immune suppression, tissue remodelling and the survival or proliferation of neighbouring malignant cells. Radiation-induced senescence and SASP signalling have been associated with accelerated glioblastoma growth in experimental models, including through effects arising from both irradiated tumour cells and non-malignant astrocytes within the irradiated microenvironment [18,19]. Evidence of therapy-induced senescence in human tumour samples further supports its potential clinical relevance, although its prevalence and phenotype vary substantially between treatment and tumour contexts [20].

These observations have stimulated interest in senotherapy as an adjunct to RT. Senolytic agents aim to selectively eliminate senescent cells by targeting acquired survival dependencies, whereas senomorphic approaches seek to suppress detrimental SASP signalling without necessarily inducing cell death. A potential sequential strategy would use RT to induce tumour damage and senescence, followed by appropriately timed senotherapy to remove persistent senescent cells or limit their pro-tumour effects. In preclinical glioblastoma models, navitoclax-mediated clearance of senescent astrocytes attenuated tumour growth, while more recent work identified cellular inhibitor of apoptosis protein 2 (cIAP2) as a survival dependency in radiation-induced senescent glioma cells and demonstrated that the second mitochondria-derived activator of caspases (SMAC) mimetic birinapant could reduce post-RT recurrence [19,21]. However, senotherapy remains experimental. Its translation will require robust biomarkers of clinically relevant senescence, careful treatment sequencing, and discrimination between beneficial transient senescence and persistent tumour-promoting states. The cellular origin and timing of senescence may therefore be as important as its presence when selecting patients for future RT-senotherapy combinations.

### 2.3. DDR Inhibition as a Strategy to Enhance Radiotherapy

Given the central role of DNA damage in mediating the anti-tumour effects of radiotherapy, pharmacological inhibition of DDR pathways has emerged as a compelling strategy to enhance radiosensitivity and exploit tumour-specific repair dependencies [9]. Beyond augmenting tumour cell kill, unresolved DNA damage may also shape downstream immune responses, raising the possibility that DDR inhibition could potentiate both the cytotoxic effects of RT and its immunogenic effects; that is, its capacity to promote immune recognition and activation against tumour cells [22].

#### 2.3.1. PARP Inhibition: Proof-of-Principle

PARP inhibitors were the first DDR-targeted therapies to achieve successful clinical translation and remain standard-of-care in BRCA-mutant ovarian, breast and prostate cancers. Their success provided proof-of-principle for exploiting tumour-specific DNA repair vulnerabilities through synthetic lethality [15]. This success generated considerable interest in extending PARP inhibition beyond systemic therapy and into combination with RT. Ionising radiation induces substantially more SSBs than DSBs [4]. PARP inhibition impairs repair of these lesions, promoting persistence of DNA damage and conversion of SSBs into replication-associated DSBs during cell division. Importantly, this radiosensitising mechanism is distinct from classical synthetic lethality in HR-deficient tumours and may therefore extend beyond BRCA-mutant cancers. By increasing the burden of unrepaired radiation-induced DNA damage, PARP inhibition enhances tumour cell kill and provides a strong biological rationale for combination with RT [15].

Despite promising preclinical rationale, clinical translation of PARP–RT combinations has proven challenging, with several studies limited by treatment-related toxicity, including mucosal toxicity in head and neck cancers [23] and oesophagitis in thoracic malignancies [24]. However, the phase I PARADIGM study demonstrated encouraging tolerability of olaparib combined with hypofractionated RT in patients with glioblastoma unsuitable for radical chemoradiotherapy, suggesting that acceptable therapeutic windows may exist in selected clinical settings and tumour types [25]. Nonetheless, the overall clinical impact of PARP–RT combinations has been modest, prompting increasing interest in next-generation DDR targets with broader radiosensitising and immunomodulatory potential.

#### 2.3.2. ATR Inhibition: Next-Generation Radiosensitisation

In recent years, ATR has emerged as a particularly promising target for enhancing the effects of RT. ATR plays a central role in the DDR by sensing replication stress, including stalled replication forks and regions of single-stranded DNA generated during DNA repair, and coordinating cell-cycle arrest through ATR-CHK1 signalling at the S and G2/M checkpoints. This function is particularly relevant in malignant cells harbouring defective G1 checkpoint control, most commonly through TP53 dysfunction, rendering tumours increasingly reliant on ATR-mediated cell-cycle regulation for survival following genotoxic stress. Consequently, ATR inhibition (ATRi) has attracted significant interest as a radiosensitisation strategy capable of promoting persistent DNA damage, checkpoint abrogation, and mitotic catastrophe following ionising radiation [16].

Importantly, the therapeutic implications of ATR inhibition may extend beyond enhanced tumour cell kill. Accumulating evidence suggests that unresolved DNA damage following ATR inhibition promotes the generation of cytosolic DNA and micronuclei, enhancing inflammatory signalling and innate immune activation [26,27]. Preclinical studies have demonstrated increased interferon signalling, immune cell infiltration, and TME remodelling following combined ATRi and RT. In murine head and neck cancer models, ATR inhibition combined with RT increased activation of T cells co-expressing natural killer group 2A (NKG2A) and programmed cell death protein 1 (PD-1) and expanded T-cell receptor repertoire diversity. Furthermore, dual NKG2A and programmed death-ligand 1 (PD-L1) blockade following ATRi–RT induced durable anti-tumour responses, highlighting the potential for rational combinations of DDR inhibition, RT, and immune checkpoint blockade [28].

However, emerging evidence also underscores the complexity of the DDR–immune interface. The immunological consequences of ATR inhibition appear highly dependent on treatment scheduling, with inflammatory signalling and T-cell proliferation observed following cessation of ATR inhibition, while prolonged exposure may paradoxically suppress immune activation [29]. Such findings emphasise the importance of temporal optimisation when designing combination immuno-radiotherapy strategies and may partly explain the discrepancy between strong mechanistic rationale and more modest clinical success observed across some RT–immunotherapy paradigms.

Encouragingly, early clinical translation has demonstrated promising signals of feasibility and efficacy. In the phase Ib PATRIOT Part C trial, ceralasertib combined with palliative RT was well tolerated and associated with durable responses, including complete responses in selected patients. Notably, increased peripheral T-cell and natural killer cell activation was observed over the course of treatment, providing early human evidence that the immunomodulatory effects observed preclinically may translate into clinical benefit [30]. Although considerable challenges remain, ATR inhibition represents one of the most compelling examples of how DDR targeting may evolve beyond radiosensitisation towards a broader paradigm of immunogenic radiosensitisation.

#### 2.3.3. Emerging DDR Targets Beyond PARP and ATR

Beyond PARP and ATR, a number of additional DDR targets are being explored to enhance radiosensitivity and exploit tumour-specific repair dependencies. DNA-PK, a central mediator of NHEJ, represents a particularly attractive radiotherapy-specific target given its critical role in repairing radiation-induced DSBs [31]. Pharmacological inhibition of DNA-PK impairs DSB repair and prolongs radiation-induced genomic instability, thereby enhancing tumour cell kill. In a phase I study, peposertib was tolerable at biologically relevant exposure when combined with palliative RT. However, when added to curative-intent cisplatin chemoradiotherapy, the tolerable dose produced insufficient drug exposure and a recommended phase II dose was not established, illustrating the difficulty of achieving tumour radiosensitisation without intensifying normal-tissue toxicity [32,33].

ATM, an apical regulator of DSB sensing and G1/S checkpoint signalling, has similarly emerged as a promising radiosensitisation target [34]. Preclinical studies suggest ATM inhibition may potentiate radiation-induced cytotoxicity through impaired DNA repair and checkpoint dysfunction, although clinical translation remains limited by the need for biomarker-driven patient selection and optimisation of the therapeutic window [35]. The brain-penetrant inhibitor AZD1390 has demonstrated radiosensitising activity in preclinical glioma and brain-metastasis models and measurable central nervous system exposure in humans, providing a rationale for its early-phase evaluation with cranial RT. However, published clinical outcome data remain limited, and the optimal biomarker strategy is uncertain: loss of ATM may confer intrinsic radiation sensitivity, but a tumour lacking functional ATM may not necessarily remain susceptible to pharmacological ATM inhibition [36,37].

Downstream checkpoint kinases, including WEE1 and CHK1, are also under active investigation, particularly in TP53-mutant tumours characterised by defective G1 checkpoint control and increased dependence on G2/M checkpoint regulation. Inhibition of these pathways may enhance radiosensitivity by forcing mitotic progression despite unresolved DNA damage, promoting mitotic catastrophe and tumour cell death [38]. Early-phase clinical studies have evaluated adavosertib or the CHK1 inhibitor prexasertib with RT and chemoradiotherapy. These studies demonstrate that checkpoint inhibition can be delivered with RT in selected settings, but haematological and mucosal toxicities have limited some combinations, and efficacy has not been established. For example, adavosertib with cranial RT was tolerable in paediatric diffuse intrinsic pontine glioma but did not improve survival relative to historical controls, while prexasertib could not be combined safely at the tested dose with cisplatin chemoradiotherapy in locally advanced head and neck cancer [39,40].

The relative promise of these strategies reflects not only their ability to radiosensitise tumour cells, but also the selectivity, therapeutic window and clinical context in which this can be achieved. PARP inhibitors have the greatest clinical maturity as systemic anti-cancer agents, but their combination with RT has frequently been constrained by overlapping normal-tissue and haematological toxicity, particularly when extensive normal-tissue volumes are irradiated [24,41,42]. ATR inhibition may offer broader tumour selectivity because cancers with high replication stress, defective G1 checkpoint control or loss of ATM signalling become particularly dependent on the ATR-CHK1 axis following radiation-induced damage [30]. Its effects may also extend to immune activation, although these are strongly schedule-dependent [29,30]. DNA-PK inhibition provides perhaps the most direct means of preventing repair of radiation-induced DSBs, but the importance of NHEJ in irradiated normal tissues creates a potentially narrow therapeutic window [32]. ATM and WEE1/CHK1 inhibition may be most applicable in molecularly selected tumours with defined checkpoint dependencies, but candidate biomarkers and optimal treatment schedules remain insufficiently validated [43]. Among these approaches, ATR inhibition appears particularly promising, but no DDR inhibitor has yet demonstrated sufficient prospective clinical utility with RT to guide routine treatment selection. The candidate biological contexts, current RT-specific evidence and principal translational limitations of these approaches are summarised in Table 1.

**Table 1:** Translational status of DDR-targeted radiosensitisation strategies.

Target		Candidate Biological Context	Current RT-Specific Evidence	Principal Translational Limitation
**HR deficiency**				
	**PARP**	HR deficiency may enhance tumour selectivity in particular contexts, but radiosensitisation is not restricted to HR-deficient tumours and no RT-specific selection biomarker is validated.	Multiple phase I and phase II studies. Some combinations are feasible, but randomised studies have not demonstrated clinical benefit [23,24,25,41,42].	Variable normal-tissue and haematological toxicity, including potential late toxicity; limited efficacy despite strong preclinical rationale.
**Defective G1 checkpoint**				
	**ATR**	High replication stress, ATM loss or reduced signalling, and defective G1 checkpoint control are candidate contexts; none is prospectively validated for RT selection.	Phase I human RT evidence, including pharmacodynamic and immune-activation signals; comparative efficacy remains unproven [26,28,29,30].	Schedule-dependent effects, haematological and normal-tissue toxicity, and absence of a validated selection biomarker.
	**CHK1**	High replication stress or defective G1 checkpoint control; no validated RT-selection biomarker.	Limited phase I evidence: cisplatin–RT was intolerable at the tested dose, whereas cetuximab–RT was feasible [39].	Prominent haematological and mucosal toxicity and limited clinical evidence.
	**WEE1**	TP53/G1-checkpoint dysfunction and high replication stress are candidate contexts but are not validated selection biomarkers.	Multiple phase I RT or chemoradiotherapy studies demonstrate feasibility in selected settings; efficacy remains unestablished [40,43].	Haematological and mucosal toxicity, combination intensity and uncertain molecular selection.
**DNA-PK–mediated NHEJ and DSB signalling**				
	**DNA-PK**	No validated biomarker; complementary repair defects and highly conformal RT may provide greater tumour selectivity.	Phase I data with palliative RT. With cisplatin chemoradiotherapy, a tolerable dose produced insufficient exposure and no recommended phase II dose was established [32,33].	Essential role of NHEJ in irradiated normal tissues, potentially narrow therapeutic window and difficulty combining with intensive chemoradiotherapy.
	**ATM**	ATM-expressing tumours; central nervous system tumours are of particular interest for brain-penetrant inhibitors. No predictive biomarker is validated.	Preclinical radiosensitisation and human central nervous system pharmacokinetic evidence; therapeutic RT evaluation remains early phase [36,37].	No published clinical efficacy evidence, uncertain molecular selection and potential normal-tissue radiosensitisation.

Rows are grouped and colour-coded to match the targets in Figure 1A–C. DDR, DNA damage response; DSB, double-strand break; HR, homologous recombination; NHEJ, non-homologous end joining; RT, radiotherapy.

### 2.4. From DNA Repair to Immune Activation: The DDR–Immunity Interface

Increasing evidence suggests that the biological consequences of radiation-induced DNA damage extend beyond direct tumour cell kill, positioning the interplay between DDR and immune activation as a central molecular determinant of radiotherapy response [44]. Radiation-induced DNA damage and incomplete repair can result in the accumulation of cytosolic DNA, including chromosomal fragments sequestered within micronuclei following aberrant mitosis. These structures act as potent danger-associated signals capable of stimulating innate immune responses, providing a mechanistic link between genotoxic stress and anti-tumour immunity [45].

Importantly, the immunological effects of RT appear highly dose-dependent. Up to a threshold, ionising radiation promotes type I interferon signalling, dendritic cell (DC) recruitment, and activation of anti-tumour T-cell responses, processes associated with improved immunotherapy efficacy. However, preclinical studies suggest that higher single-fraction radiation doses may paradoxically suppress this immune response through induction of three-prime repair exonuclease 1 (TREX1), a cytosolic DNA exonuclease that degrades cytosolic DNA and attenuates downstream immune signalling. Such findings may partly explain the disappointing results observed in several radiotherapy–immune checkpoint inhibitor trials and highlight the importance of optimising radiation dose and fractionation to maximise immunogenicity and systemic anti-tumour responses [46]. Collectively, these observations position DDR not merely as a determinant of radiosensitivity, but as a critical regulator of radiation-induced anti-tumour immunity. Understanding how RT shapes immune activation therefore represents a central challenge in precision radiation oncology.

## 3. Radiation and the Immune System: From Immunogenic Cell Death to cGAS–STING

### 3.1. Immunogenic Cell Death and Antigen Presentation

Beyond direct cytotoxicity, RT is recognised to exert profound immunomodulatory effects capable of reshaping anti-tumour immune responses [47]. Ionising radiation can induce immunogenic cell death, a form of tumour cell death characterised by the release of damage-associated molecular patterns (DAMPs), including high mobility group box 1 (HMGB1), calreticulin, adenosine triphosphate (ATP), and heat shock proteins. RT-induced reactive oxygen species play an important role in facilitating this process, promoting cellular stress responses and DAMP release. Collectively, these signals can transform tumour cell death into an immunogenic event, enhancing immune recognition of malignant cells [48].

DAMP-induced activation promotes the maturation of antigen-presenting cells, particularly DCs, which engulf tumour-derived antigens. Mature DCs subsequently migrate to tumour-draining lymph nodes, where cross-presentation of tumour antigens primes cytotoxic CD8+ T cells capable of recognising and eradicating malignant cells. In this way, RT may initiate local and systemic anti-tumour immune responses extending beyond direct tumour cell kill. While DAMP-mediated antigen presentation provides one mechanism through which RT enhances anti-tumour immunity, radiation-induced cytosolic DNA sensing represents another important pathway linking genotoxic stress to immune activation [49].

### 3.2. DDR–cGAS–STING Interface

Among the pathways linking radiation-induced DNA damage to anti-tumour immunity, the cyclic GMP-AMP synthase (cGAS)–stimulator of interferon genes (STING) axis has emerged as particularly important. Cytosolic DNA generated following irradiation is sensed by cGAS, triggering production of cyclic GMP-AMP (cGAMP) and subsequent activation of STING [50]. Downstream signalling through interferon regulatory factor 3 (IRF3) and nuclear factor-κB (NF-κB) promotes type I interferon production, DC maturation, antigen presentation, and priming of tumour-specific CD8+ T cells [50,51,52].

The central role of cGAS–STING signalling in coordinating innate and adaptive immunity has made it an attractive therapeutic target. Preclinical studies demonstrate that intact STING signalling is required for optimal radiation-induced type I interferon responses and durable anti-tumour immunity, providing a strong mechanistic rationale for combining RT with immunotherapy and for the development of STING-directed therapeutic strategies [53].

However, the consequences of cGAS–STING activation depend on its magnitude, duration and cellular context. Whereas acute or transient activation can promote type I interferon production, antigen presentation and T-cell priming, persistent cytosolic DNA sensing may have paradoxical tumour-promoting effects. In chromosomally unstable tumours, recurrent micronuclear rupture can sustain tumour-cell-intrinsic cGAS–STING signalling and favour non-canonical NF-κB, endoplasmic-reticulum stress and inflammatory programmes associated with immune suppression and metastatic progression [54,55]. Prolonged downstream interferon signalling may additionally induce multigenic resistance programmes involving both immune-checkpoint and checkpoint-independent pathways, potentially contributing to T-cell dysfunction and resistance to immune checkpoint blockade [56]. These findings suggest that effective therapeutic exploitation of cGAS–STING may require appropriately timed and spatially controlled activation rather than indiscriminate or sustained pathway stimulation.

Conversely, radiation-induced cGAS–STING activity may also be attenuated by endogenous “STING checkpoints”, limiting the magnitude or duration of productive anti-tumour immunity and potentially contributing to radioresistance [57]. For example, radiation-induced interferon signalling can increase expression of YTH N6-methyladenosine RNA binding protein 1 (YTHDF1) within DCs, promoting lysosomal degradation of STING through cathepsin-mediated pathways and attenuating downstream immune activation [58]. Similarly, radiation-induced expression and cleavage of heme oxygenase-1 (HO-1) may suppress cGAS–STING signalling through disruption of STING oligomerisation and inhibition of cGAS-mediated cGAMP production [59]. Such findings suggest that cGAS–STING activation exists within a tightly regulated immune network and may partly explain the discrepancy between strong mechanistic rationale and the mixed clinical results observed with radiotherapy–immunotherapy combinations [5].

Collectively, cGAS–STING signalling provides one of the clearest mechanistic links between radiation-induced DNA damage and anti-tumour immunity, but its effects are highly dependent on signalling intensity, duration and cellular context. Both insufficient activation and persistent maladaptive signalling may constrain therapeutic benefit. Defining how radiation dose, fractionation and treatment sequencing shape these distinct immune states—and identifying which tumours retain a therapeutically productive cGAS–STING response—will therefore be important for the rational development of future radiotherapy–immunotherapy combinations.

### 3.3. Dose, Fractionation and Radiation Quality as Determinants of Immune Response

The immunological consequences of RT depend not only on total dose, but also on dose per fraction, fraction number, treated volume and sequencing with systemic therapy. Conventionally fractionated schedules may provide repeated opportunities for tumour-antigen release and immune priming, but can also expose circulating and intratumoural lymphocytes to repeated irradiation. Hypofractionated or stereotactic schedules deliver greater damage per fraction and may enhance immunogenic cell death, antigen release and inflammatory signalling; however, uniformly ablative doses may also induce TREX1, damage tumour vasculature and deplete immune cells within the irradiated volume. Direct preclinical comparisons confirm that conventional fractionation, moderate hypofractionation and single-fraction irradiation generate distinct lymphoid and myeloid responses, but the preferred regimen varies between tumour models and therapeutic combinations [46,60,61]. Treatment sequencing is similarly context-dependent: the optimal timing of immune modulation relative to RT may differ according to whether the systemic agent acts through regulatory T-cell depletion, immune-checkpoint blockade or stimulation of antigen-activated effector cells [62]. Consequently, no universally immunogenic radiation schedule has been established, and dose-fractionation should be evaluated alongside treatment volume, tumour immune context and the mechanism of the accompanying systemic therapy.

Radiation quality provides an additional determinant of biological response. Photons and clinical proton beams are predominantly low-linear-energy-transfer (LET) modalities, although proton LET increases towards the distal end of the Bragg peak, whereas carbon ions produce substantially higher LET and denser ionisation tracks. Increasing LET generally produces more spatially clustered and complex DNA damage that is less readily repaired and may alter cell fate, micronucleus formation and downstream innate immune signalling [63]. Complex DSBs have been demonstrated in human tumours following carbon-ion irradiation, supporting the biological relevance of these differences [64]. Emerging experimental comparisons also suggest that proton and photon irradiation can generate distinct immune and microenvironmental responses and may interact differently with immune-checkpoint blockade [65]. However, these observations remain predominantly preclinical, and there is currently insufficient evidence to select a radiation modality primarily according to its anticipated immune effects.

### 3.4. Radiotherapy–Immunotherapy Combinations and the Abscopal Effect

The advent of immune checkpoint inhibitors (ICIs) has transformed the systemic treatment landscape across multiple tumour types, generating considerable enthusiasm for combining immunotherapy with RT to enhance radiation-induced anti-tumour immunity. A strong mechanistic rationale exists for RT–ICI combinations: tumour irradiation promotes antigen release, type I interferon signalling, DC activation, T-cell priming, and remodelling of the tumour microenvironment towards a more inflamed phenotype. Consequently, combining RT with immune checkpoint blockade, including inhibition of PD-1, PD-L1, and cytotoxic T-lymphocyte-associated protein 4 (CTLA-4), has emerged as an attractive strategy to amplify endogenous anti-tumour immune responses [5,66].

Despite compelling preclinical rationale, translation into clinical benefit has proven more challenging than initially anticipated. Although selected settings have demonstrated encouraging synergy, many clinical trials investigating RT–ICI combinations have produced mixed or disappointing results [5]. This likely reflects the complexity of radiation-induced immune modulation, in which dose, fractionation, treated volume and sequencing interact with tumour type and baseline immune contexture to determine treatment response [5,67]. These variables form part of a broader and highly dynamic interaction between RT and the tumour immune microenvironment.

An intriguing and biologically important aspect of the radiotherapy–immune interaction is the abscopal effect, whereby tumour regression occurs at sites distant from the irradiated field following local RT. Although rare and clinically unpredictable, the abscopal effect provides compelling proof-of-principle that RT can induce systemic anti-tumour immunity. Early clinical evidence emerged from reports of metastatic melanoma patients treated with RT and immune checkpoint blockade demonstrating regression of non-irradiated lesions alongside immunological correlates of response [68].

In the era of RT–ICI combinations, considerable interest has focused on strategies to deliberately augment systemic immune activation and increase the likelihood of abscopal responses in metastatic disease. Experimental approaches include combining ICIs with single-site or multi-site irradiation, ablative or sub-ablative RT, and low-dose (<1 Gy) RT intended to induce complementary immunomodulatory effects within the tumour microenvironment [5]. Such approaches have led to the proposed concept of a “radscopal effect,” which is distinct from the conventional abscopal effect and describes the use of low-dose irradiation to enhance the systemic efficacy of immunotherapy in combination with focal higher-dose RT [67]. However, despite promising preclinical rationale, these strategies remain largely investigational and require further mechanistic and clinical validation.

The unpredictability of durable systemic responses despite strong mechanistic rationale underscores the complexity of radiation-induced immune modulation. Increasing evidence suggests that the tumour microenvironment plays a critical role in determining whether RT promotes effective anti-tumour immunity or, conversely, reinforces immune suppression and treatment resistance [5].

### 3.5. The Tumour Microenvironment as an Immune Modulator

Although RT can stimulate anti-tumour immunity, it can also induce immunosuppressive processes that limit sustained tumour control. Increasing evidence suggests that radiation induces dynamic remodelling of the TME, simultaneously promoting immune activation while also facilitating adaptive resistance through immune exhaustion, recruitment of suppressive immune populations, and escape from immune surveillance. Consequently, the balance between immunostimulatory and immunosuppressive effects within the irradiated TME may ultimately determine whether RT generates durable anti-tumour immunity or treatment resistance [69].

Metabolic conditions within the TME provide an additional link between radioresistance and immune suppression. Hypoxia reduces the oxygen-dependent fixation of radiation-induced DNA damage, diminishing the biological effectiveness of low-LET radiation [70]. Glycolytic tumours may also generate lactate-rich, acidic and nutrient-depleted microenvironments that impair T-cell and natural killer-cell function and restrict immune-cell metabolism [71,72]. These spatially heterogeneous conditions may therefore influence both tumour radiosensitivity and radiation-induced immune responses. Although metabolic imaging and therapeutic modulation of hypoxia or tumour metabolism are attractive precision-RT strategies, validated biomarkers capable of guiding radiation prescription remain lacking.

The importance of biomarker context is illustrated by exploratory translational analyses of JAVELIN Head and Neck 100, a phase III trial in which the addition of avelumab to definitive chemoradiotherapy did not improve progression-free survival in an unselected population. Subsequent profiling of trial specimens identified tumour ecosystem features associated with differential outcomes, including immune-inflamed phenotypes and myeloid-, neutrophil- and intratumoral-bacteria-associated states. These analyses provide hypotheses about mechanisms of sensitivity and resistance but do not establish prospectively validated predictive biomarkers for selecting patients for RT–ICI combinations. Further validation in independent cohorts and biomarker-stratified trials is therefore required [73].

Accordingly, increasing attention has focused on therapeutic strategies capable of reprogramming the post-radiation tumour microenvironment to overcome adaptive resistance. Preclinical work has begun to characterise key drivers of resistance to combined RT and immune checkpoint inhibition, identifying suppressive immune signalling pathways that may limit durable anti-tumour responses. In murine bladder and prostate cancer models resistant to RT and ICI therapy, the addition of an agonistic anti-CD40 monoclonal antibody to RT enhanced interferon-γ signalling, promoted T-helper 1 (Th1) immune pathways, and increased infiltration of cytotoxic CD8+ T cells within the tumour microenvironment. Notably, this inflammatory remodelling was accompanied by activation of compensatory CTLA-4 signalling, providing mechanistic rationale for further combinatorial immune targeting. Importantly, therapeutic reprogramming of the TME resulted in durable tumour clearance in otherwise resistant models, suggesting that future progress may depend less on intensifying treatment and more on rationally reshaping radiation-induced immune states. Such approaches raise the possibility of converting immunologically “cold” tumours into more treatment-responsive phenotypes [74].

Emerging evidence further suggests that radiation-induced immune remodelling may, in some contexts, paradoxically facilitate immune escape and metastatic progression. While SBRT achieves excellent local control in oligometastatic disease and may suppress metastatic seeding, recent translational work has highlighted potentially adverse systemic immune consequences of irradiation. Mechanistically, radiotherapy may upregulate amphiregulin (AREG), an epidermal growth factor receptor ligand implicated in monocyte differentiation towards immunosuppressive macrophage phenotypes. In preclinical models, this shift was associated with enhanced metastatic progression and inferior survival, suggesting that radiation-induced alterations within the tumour–immune ecosystem may simultaneously promote local tumour control while limiting durable systemic responses. Although preliminary, such findings reinforce the complexity of radiation-induced immune modulation and identify AREG as a potential biomarker and therapeutic target for improving the efficacy of metastasis-directed therapies [75]. Collectively, these observations demonstrate that the immunological consequences of RT depend on tumour biology, radiation dose and fractionation, treated volume and spatial dose distribution.

## 4. Spatially Tailored Radiotherapy to Harness the Microenvironment

**Figure 2:**
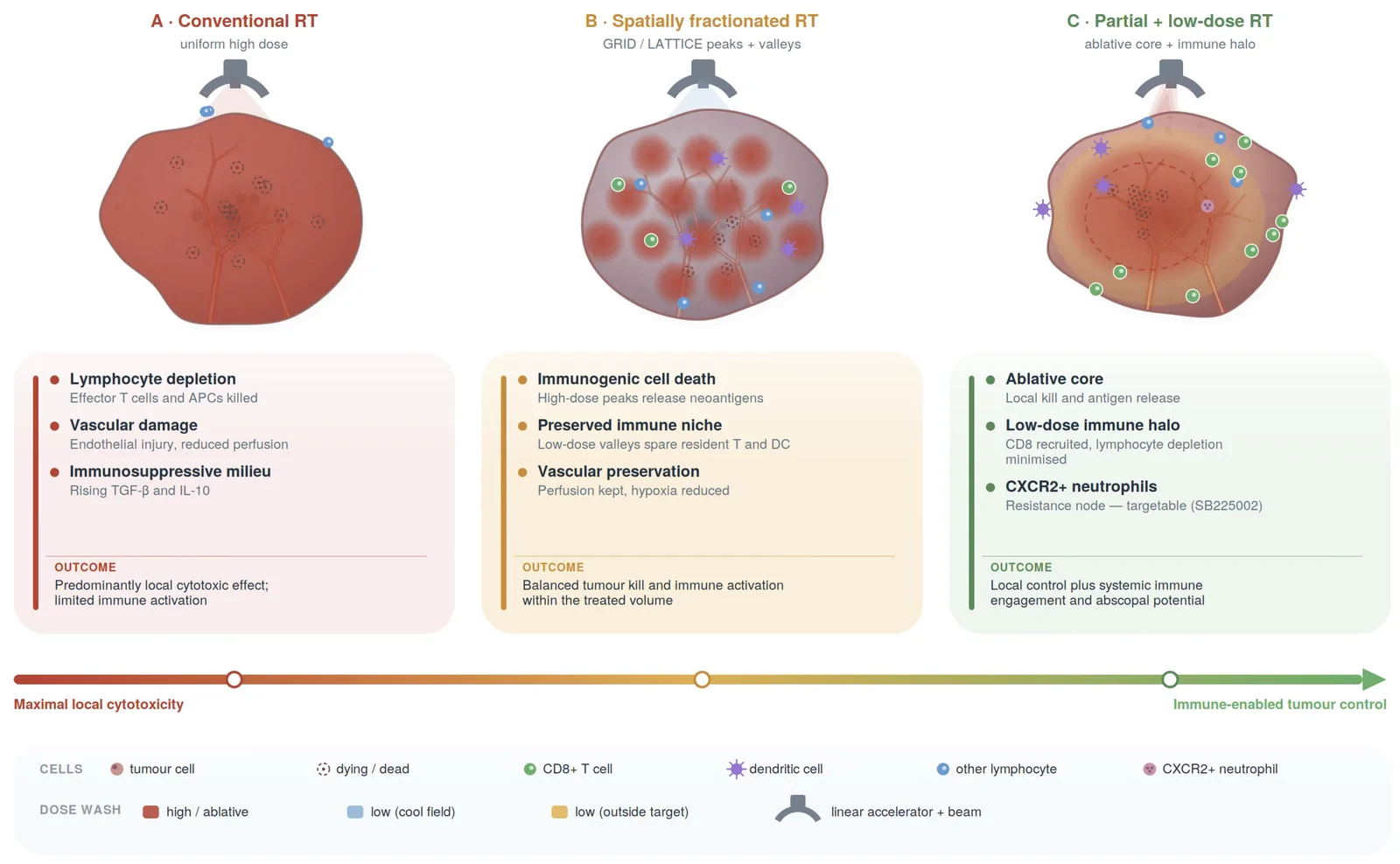
Spatially tailored RT strategies and their effects on the TME. (**A**) Conventional RT delivers a uniform high dose across the target volume, achieving effective local cytotoxicity but also promoting lymphocyte depletion, vascular injury and an immunosuppressive TME. (**B**) SFRT, including GRID and LATTICE techniques, delivers heterogeneous high-dose regions interspersed with lower-dose areas, promoting immunogenic cell death while preserving immune cell niches and vascular integrity. (**C**) Partial tumour irradiation combined with a low-dose peripheral immune-modulating field represents an emerging strategy that concentrates ablative doses within the tumour core while preserving surrounding immune and stromal components, facilitating immune cell recruitment, reducing radiation-induced lymphocyte depletion and potentially enhancing systemic anti-tumour immune responses.

While these biological insights identify new therapeutic targets, they also raise a broader question: should radiation itself be delivered differently to maximise immune activation? Growing interest has focused on whether spatially tailored radiotherapy approaches may better preserve immune function, enhance systemic anti-tumour responses, and improve synergy with immunotherapy. Such strategies challenge the conventional paradigm of treating the entire tumour uniformly, instead exploring whether variable dose delivery, or even deliberate under-treatment of selected regions, may more effectively exploit tumour–immune interactions, as demonstrated in Figure 2 [76].

### 4.1. Spatially Fractionated and Heterogeneous Intratumour Irradiation

One emerging strategy is spatially fractionated radiotherapy (SFRT), also termed heterogeneous intratumour irradiation, in which selected regions of a tumour receive high-dose radiation while the remainder is treated with a substantially lower dose [77]. Techniques including GRID [78], LATTICE [79], and other heterogeneous dose-painting approaches are based on the premise that not all tumour regions necessarily require identical radiation exposure to achieve effective tumour control. Instead, strategic dose heterogeneity may permit simultaneous tumour cytoreduction and immune preservation [77].

The mechanistic rationale for SFRT is increasingly compelling. Focal high-dose regions may induce profound tumour cell death, immunogenic cell death, and antigen release, while lower-dose regions may preserve vascular integrity and immune cell populations within the TME, potentially facilitating ongoing immune infiltration and anti-tumour activity following irradiation. In contrast, irradiation of the entire tumour at uniformly ablative doses may unintentionally deplete lymphocytes and antigen-presenting cells required to sustain effective immune responses [80]. Preclinical evidence increasingly supports the concept that non-uniform dose delivery may promote more favourable immune remodelling than conventional homogeneous irradiation [81].

Recent studies have demonstrated that spatially heterogeneous irradiation can reshape the tumour immune landscape and enhance systemic immune activation, further supporting the hypothesis that selective high- and low-dose regions may work synergistically to optimise both local control and anti-tumour immunity [81]. However, despite growing enthusiasm, substantial uncertainties remain. The optimal spatial dose distribution, fractionation schedule, and relative proportion of tumour requiring ablative versus immunomodulatory doses remain undefined, and whether preservation of immune function outweighs the benefits of conventional full-dose irradiation remains unclear [82]. Accordingly, SFRT remains an intriguing but highly experimental strategy requiring further mechanistic and clinical validation.

### 4.2. Partial Tumour Irradiation and Low-Dose Immune Modulation

A related but conceptually distinct strategy involves deliberate partial tumour irradiation and low-dose radiotherapy aimed at immune modulation rather than maximal tumour cytotoxicity. Rather than treating the entire tumour uniformly, emerging evidence suggests that combining focal high-dose radiation with lower-dose treatment of selected tumour regions may better harness immune-mediated anti-tumour responses. Recent preclinical work demonstrated that partial tumour irradiation combining high-dose and low-dose RT enhanced anti-tumour responses in murine colorectal and breast cancer models when combined with immune checkpoint blockade. Mechanistically, high-dose RT promoted tumour cell kill, while lower-dose regions enhanced infiltration of interferon-activated cytotoxic CD8+ T cells. Importantly, resistance appeared partly mediated through C-X-C chemokine receptor type 2 (CXCR2)-driven neutrophil infiltration, with addition of the CXCR2 antagonist SB225002 further improving tumour control and survival. These findings suggest that deliberate manipulation of intratumour dose gradients may augment anti-tumour immunity while identifying novel therapeutic vulnerabilities within the TME [81].

Recent clinical and mechanistic evidence has also raised the possibility that low-dose irradiation outside the tumour may modulate systemic treatment responses. Chen et al. reported associations between incidental intestinal low-dose irradiation and improved outcomes across retrospective patient cohorts, supported by mechanistic experiments and an early interventional phase II proof-of-concept study in patients with immunotherapy-resistant metastatic cancer [83]. Although these findings are provocative, intestinal low-dose irradiation remains an experimental strategy whose efficacy, safety, optimal dose and patient-selection criteria require confirmation in prospective randomised trials.

Additional preclinical evidence further supports a role for low-dose RT in enhancing immunotherapy responses through mechanisms including ferroptosis induction [84], tumour microenvironment remodelling, augmentation of anti-CTLA-4 activity [85], and enhancement of PD-L1 blockade with associated abscopal responses [86]. Collectively, these studies provide a hypothesis-generating extension of spatially tailored RT, in which selected low-dose or partial-volume exposures are investigated for their potential biological and immunological effects rather than regarded solely as unintended dose. These approaches remain experimental and should not presently be interpreted as alternatives to established principles of target coverage and normal-tissue protection.

## 5. Biological Personalisation of Radiotherapy: Toxicity, Genomics and Host Factors

**Figure 3:**
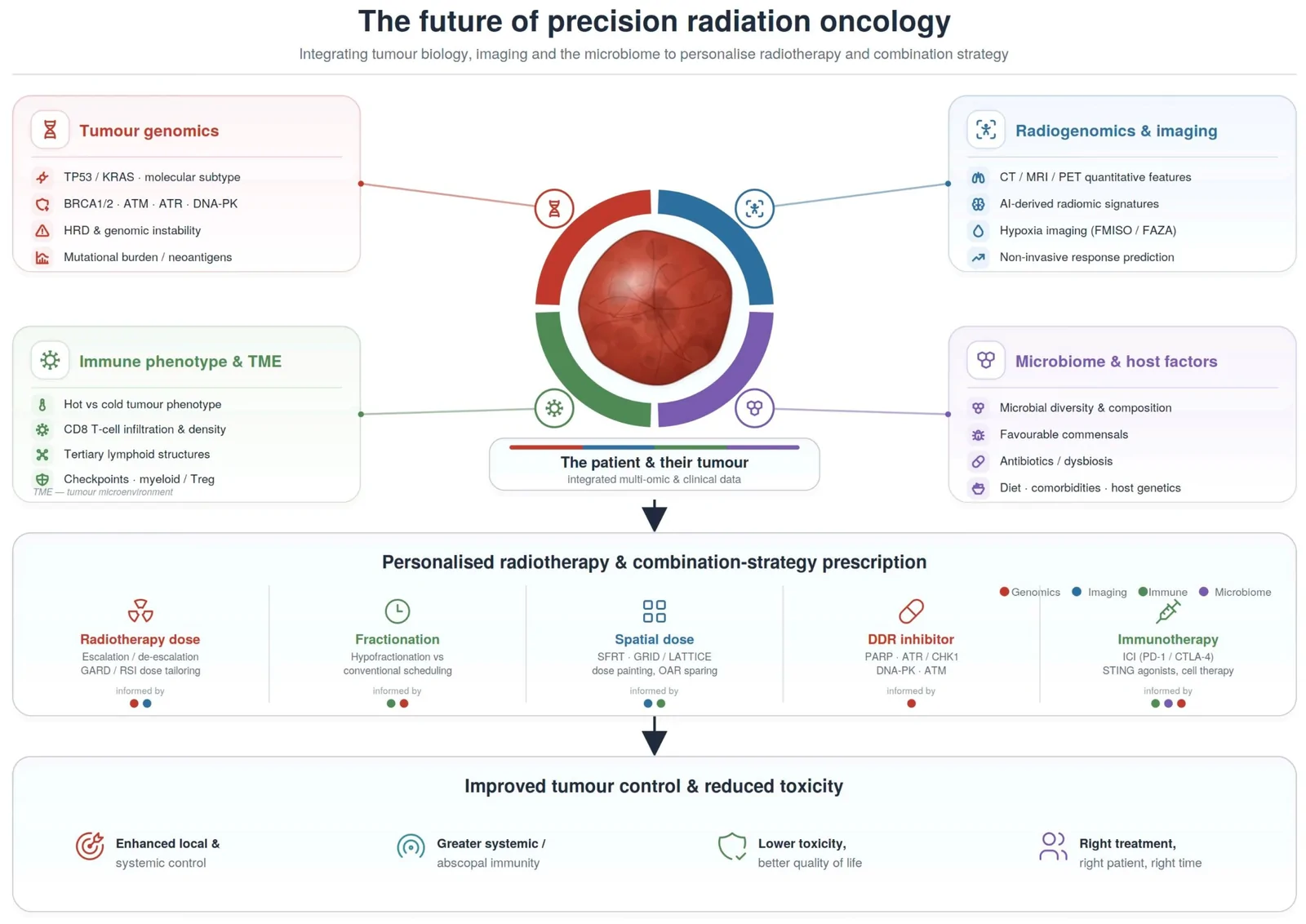
Conceptual framework for biological personalisation of radiotherapy. Candidate genomic, imaging, immune, microenvironmental and host factors may contribute to models of tumour response and normal-tissue toxicity. Their use in treatment selection requires reproducible measurement, integration into a prespecified predictive model and prospective demonstration of clinical utility.

As radiation oncology moves beyond anatomical precision towards biological individualisation, increasing attention has focused on interpatient variation in both tumour response and normal-tissue toxicity. Precision radiotherapy therefore requires integration of tumour radiosensitivity with the susceptibility of surrounding healthy tissues, alongside imaging phenotypes, immune context and host factors. Emerging approaches seek to combine these complementary sources of information to guide treatment selection, radiation dose and fractionation, spatial dose distribution and rational combinations with systemic therapy, while preserving the therapeutic ratio [87,88]. The candidate biological inputs and translational pathway discussed in this section are summarised in Figure 3.

The extent to which tumour subtype or molecular alteration currently determines an RT-containing regimen varies considerably. At the most clinically established level, molecular classification can define disease entities for which particular multimodality regimens are recommended; for example, IDH-mutant, 1p/19q-codeleted oligodendroglioma is treated using an RT–alkylating-chemotherapy strategy [89], while MGMT promoter methylation helps determine the likely benefit of adding temozolomide to hypo-fractionated RT in older patients with glioblastoma [90]. A second category comprises biologically selected de-intensification strategies under prospective evaluation, including reduced-dose RT in favourable-risk, human papillomavirus-associated oropharyngeal cancer [91]. A third, less mature category uses candidate genetic dependencies, such as homologous-recombination deficiency, TP53 dysfunction, ATM loss or high replication stress, to select PARP, ATR, WEE1 or related inhibitors for combination with RT. These examples illustrate that molecular information may influence treatment indication, systemic combination or investigational dose adaptation, but direct alteration of radiation dose or fractionation according to tumour genotype remains uncommon and generally requires prospective validation.

### 5.1. Normal-Tissue Radiosensitivity and Optimisation of the Therapeutic Ratio

The central objective of precision radiotherapy is not simply to maximise tumour response, but to optimise the therapeutic ratio by balancing tumour-control probability (TCP) against normal-tissue complication probability (NTCP). Radiation toxicity may develop during treatment or emerge months to decades later, reflecting distinct but overlapping biological processes. Acute toxicity commonly results from DNA damage, inflammatory signalling and depletion of rapidly proliferating epithelial or haematopoietic cell populations. In contrast, late effects may involve persistent DNA damage, cellular senescence, microvascular dysfunction, loss of tissue stem and progenitor cells, chronic inflammation, extracellular-matrix remodelling and fibrosis. Their incidence and severity are influenced by total dose, dose per fraction, irradiated volume and organ architecture, as well as age, comorbidity, baseline organ function, concurrent systemic therapy and individual biological susceptibility [87]. Consequently, patients receiving apparently comparable treatment plans may experience markedly different toxicities.

Current toxicity prediction relies predominantly on clinical characteristics and dose-volume parameters incorporated into NTCP models. Genomic variants and molecular signatures may provide additional information regarding DNA repair capacity, inflammatory responses and fibrotic susceptibility; however, many reported associations have shown limited replication or only modest predictive performance when added to clinical and dosimetric factors. Functional radiosensitivity assays offer a complementary approach by directly measuring an individual cellular phenotype. These include radiation-induced lymphocyte apoptosis, chromosomal aberration and micronucleus assays, comet assays, and quantification of γ-H2AX formation and resolution as a measure of DSB repair kinetics. Patient-derived cellular or organoid models may eventually provide more tissue-specific assessment. Although several assays have been associated with subsequent normal-tissue toxicity, methodological heterogeneity, uncertain thresholds, limited prospective validation and practical barriers to standardisation currently preclude their routine use for treatment selection [88]. Functional, genomic and clinical measurements should therefore be regarded as complementary components of an integrated prediction model rather than interchangeable or independently treatment-guiding biomarkers.

A toxicity biomarker becomes clinically useful only when it changes a specified treatment decision and improves net clinical benefit. Potential applications include selection of radiation modality or treatment plan, modification of dose or fractionation, avoidance or altered sequencing of radiosensitising systemic therapy, and intensified surveillance or preventive intervention for patients at elevated risk. Toxicity may also be mitigated through established approaches including image guidance, adaptive treatment, organ-at-risk dose constraints and, in selected settings, proton therapy or other modalities that reduce normal-tissue exposure [87]. However, dose reduction or treatment omission based on an insufficiently validated toxicity marker could compromise tumour control. Prospective evaluation must therefore assess both TCP and NTCP and demonstrate that biomarker-guided adaptation produces a clinically meaningful improvement in the overall therapeutic ratio. Integrating functional radiosensitivity, genomic information and clinical-dosimetric modelling provides a plausible framework for this objective, but remains an important research priority rather than established routine practice [88].

Throughout this review, it is important to distinguish prognostic association from predictive and treatment-guiding utility. A prognostic biomarker is associated with outcome irrespective of the treatment received, whereas a predictive biomarker identifies differential benefit from a particular treatment, dose or fractionation strategy. For a biomarker to guide radiotherapy prescription, its analytical validity, reproducibility and incremental value over established clinical and dosimetric factors must be demonstrated, and the resulting treatment decision should be evaluated prospectively. Accordingly, associations identified in retrospective cohorts or exploratory translational analyses should generally be regarded as hypothesis-generating until their clinical utility has been confirmed.

### 5.2. Radiogenomics and Genetic Profiling of Radiosensitivity

The term radiogenomics has been used in two related but distinct ways. In imaging research, it describes the association of quantitative imaging phenotypes with underlying genomic characteristics of a tumour. In radiation oncology, it has also been used to describe the study of genomic variation—particularly germline variation—in relation to radiation response and normal-tissue toxicity [92]. Although both approaches seek to improve biological personalisation of RT, they measure different aspects of treatment response and should not be considered interchangeable.

Imaging-based radiogenomics combines features extracted from MRI, PET, CT or other imaging modalities with genomic and clinical data to characterise tumour biology non-invasively [93]. Such features may provide spatially resolved correlates of molecular heterogeneity that are not fully represented by a single tumour biopsy. For example, MRI-derived models have been investigated for the non-invasive classification of isocitrate dehydrogenase (IDH) mutation status in glioma [94,95]. However, these studies primarily demonstrate associations between imaging phenotype and tumour genotype; they do not necessarily predict differential benefit from RT or provide a validated basis for modifying radiation prescription. Translation is further limited by differences in image acquisition, segmentation and feature extraction, together with limited external validation and potential model instability across institutions.

In the second usage, radiogenomics investigates whether inherited genetic variation contributes to interpatient differences in radiation response, particularly susceptibility to normal-tissue toxicity [96]. Candidate-gene and genome-wide approaches have examined variants affecting DNA repair, inflammatory signalling and tissue responses to radiation. For example, an association between an XRCC1 polymorphism and late toxicity following breast radiotherapy has been reported [97]. However, individual common variants generally have small effect sizes, and reported associations require replication in adequately powered, ancestrally diverse cohorts with standardised toxicity phenotyping and detailed dosimetric adjustment. Germline genomic information may ultimately contribute to multivariable normal-tissue complication models, but it has not yet demonstrated sufficient prospective clinical utility to guide routine dose or fractionation decisions. Thus, both forms of radiogenomics remain promising components of precision RT, but their clinical value depends on demonstrating that they improve prediction beyond established clinical and dosimetric factors and lead to a defined, prospectively validated treatment decision.

### 5.3. GARD: Towards Genomically Adjusted Radiation Prescription

An important application of biological stratification is the use of genomic biomarkers to individualise radiation dose. The genomic-adjusted radiation dose (GARD) framework represents one of the most extensively studied attempts to integrate tumour gene expression with the physical radiation dose. GARD combines the radiosensitivity index with the linear-quadratic model to estimate the biological effect of a prescribed dose. Retrospective and pooled cohort analyses have reported associations between higher GARD values and improved outcomes following RT and have examined statistical interactions between GARD and receipt of RT [98,99]. These findings support further evaluation of biologically informed dose modelling but do not, by themselves, demonstrate that GARD-guided dose adaptation improves patient outcomes.

GARD has subsequently been evaluated across several tumour types. In nasopharyngeal carcinoma, Chiang et al. reported substantial interpatient variation in GARD despite standardised treatment, with higher GARD values associated with improved locoregional control [100]. Similarly, a retrospective analysis in locally advanced rectal cancer reported associations between GARD and treatment response and disease-free survival despite a uniform prescribed dose [101]. These studies provide further evidence that the biological effect of a given physical dose may vary between tumours. However, they should be regarded as retrospective validation of association rather than evidence that GARD can yet be used to select or modify an individual patient’s radiation prescription.

GARD therefore remains investigational. Important limitations include uncertainty regarding its direct predictive value for locoregional control, limited prospective validation, challenges in applying the model to altered fractionation, and failure to account for spatial intratumour heterogeneity [102,103]. Its reproducibility across tumour types, incremental value over established clinical models, and safety when used to inform dose escalation or de-escalation require evaluation in prospective trials in which the biomarker is linked to a predefined treatment decision. Nevertheless, GARD provides an important conceptual framework for investigating genomically informed radiation prescription, while illustrating the distinction between an outcome-associated biomarker and one with demonstrated treatment-guiding clinical utility.

### 5.4. Gut Microbiome and Antibiotic Exposure as Candidate Modulators of RT–ICI Response

Beyond tumour-intrinsic genomics, host factors may influence response to immunotherapy and potentially to RT–ICI combinations. The gut microbiome contributes to systemic immune regulation, and clinical studies have reported associations between microbial composition, antibiotic exposure and outcomes following immune checkpoint inhibition [104,105]. Greater microbial diversity and the relative abundance of particular taxa have been associated with favourable ICI outcomes in some cohorts, whereas dysbiosis has been linked to resistance [104,105]. However, the taxa implicated have varied between studies, and most clinical evidence concerns patients receiving ICIs rather than RT–ICI combinations. Microbiome features should therefore be regarded as candidate biomarkers requiring prospective validation, rather than established predictors of treatment response.

Antibiotic exposure is a clinically relevant potential modifier of this relationship. By disrupting microbial composition, antibiotics may alter antigen presentation and systemic effector T-cell responses. Observational studies have associated antibiotic exposure around the initiation of ICIs with inferior outcomes [104,105], but these findings do not establish causality: infection, comorbidity, disease burden and treatment indication may themselves influence prognosis. Antibiotic exposure should therefore not currently be used as an independent basis for treatment selection. Conversely, selective targeting of tumour-associated bacteria may offer a more directed therapeutic strategy. In preclinical colorectal cancer models, liposomal antibiotic targeting of Fusobacterium nucleatum generated microbial neoantigens, increased anti-tumour immune responses and enhanced immunotherapy activity [106]. This approach remains mechanistic and preclinical rather than clinically validated.

For precision radiation oncology, these observations suggest that host microbial factors may contribute to the biological context in which radiation-induced immune signalling occurs. Nevertheless, there is currently insufficient evidence to incorporate microbiome composition into routine selection of RT–ICI combinations or radiation prescription. Prospective studies will need to define reproducible microbial signatures, control for diet, geography, medication exposure and other confounders, and demonstrate that microbiome-informed interventions improve outcomes. Microbiome profiling and manipulation therefore remain experimental components of precision RT rather than treatment-guiding tools.

Translating these heterogeneous inputs into precision RT requires more than their inclusion in a conceptual framework. A candidate biomarker must first be measured reproducibly and shown to add information beyond established clinical, pathological and dosimetric variables. It must then be incorporated into a prespecified predictive model linked to a defined treatment decision—for example, whether to use RT, alter dose or fractionation, modify the spatial dose distribution, select a systemic combination or introduce additional toxicity-mitigation measures. The complete biomarker-model-decision pathway must subsequently undergo independent validation and prospective assessment of clinical utility. Because no validated method currently exists for weighting all the molecular, imaging, immune and host factors illustrated in Figure 3, the figure should be interpreted as a conceptual research framework rather than an immediately deployable clinical algorithm.

## 6. Conclusions

Radiotherapy is moving beyond anatomical precision and uniform population-based prescription towards treatment informed by tumour biology, immune context and normal-tissue susceptibility. However, precision RT should not be equated solely with increasing tumour dose or adding radiosensitising agents. Its central objective is to improve the therapeutic ratio by identifying which patients require treatment intensification, which may benefit from altered dose, fractionation or systemic combinations, and which are at greatest risk of toxicity. Molecular association alone is insufficient: a biomarker becomes clinically useful only when it reproducibly informs a defined treatment decision and improves patient outcomes prospectively.

In our view, the most credible near-term opportunity lies in biomarker-enriched development of rational RT–drug combinations rather than immediate genome-directed alteration of radiation dose. Among DDR targets, ATR inhibition appears particularly promising because radiation-treated tumours with high replication stress, defective G1 checkpoint control or loss of ATM signalling may become dependent on the ATR-CHK1 axis. Nevertheless, its therapeutic window, optimal scheduling and selection biomarkers remain unresolved, and no DDR inhibitor currently has sufficient prospective evidence to guide routine use with RT. Similarly, the future of RT–ICI combinations is likely to depend less on their indiscriminate application and more on disease-specific selection, treatment sequencing, dose and fractionation, and the pre-existing immune state. Transient activation of pathways such as cGAS–STING may support anti-tumour immunity, whereas sustained signalling and radiation-induced immunosuppression illustrate why mechanistic context and timing are critical.

Other approaches remain further from clinical implementation. Spatially fractionated and partial-tumour irradiation warrant prospective investigation, particularly where conventional homogeneous irradiation is limited by tumour volume or normal-tissue constraints. However, deliberate low-dose irradiation of non-tumour tissues, microbiome manipulation and related systemic immune-modulation strategies remain hypothesis-generating and should not displace established principles of target coverage and normal-tissue protection. GARD, imaging-based radiogenomics, germline toxicity markers and functional radiosensitivity assays provide valuable frameworks for biological stratification, but none yet justifies routine biomarker-directed dose escalation, de-escalation or treatment omission.

Progress will therefore require a disciplined translational pathway from mechanistic insight to reproducible biomarkers, a prespecified predictive model, defined treatment decisions and prospective demonstration of clinical utility. Trials should evaluate tumour-control probability and normal-tissue complication probability together, while distinguishing exploratory associations from genuinely predictive biomarkers. Precision radiation oncology will advance not through a single molecular target or universal algorithm, but through the integration of biological evidence with clinical judgement, dosimetry and technological delivery. The goal is not merely to deliver radiation more accurately, but to determine more reliably whether, when, where and how it should be delivered for each patient.

## Data Availability

No new data were created or analyzed in this study. Data sharing is not applicable to this article.

## Abbreviations

The following abbreviations are used in this manuscript:

RT	Radiotherapy
IMRT	Intensity-modulated radiotherapy
VMAT	Volumetric modulated arc therapy
SBRT	Stereotactic body radiotherapy
DDR	DNA damage response
TME	Tumour microenvironment
ATM	Ataxia telangiectasia mutated
ATR	ATM and Rad3-related
CHK1	Checkpoint kinase 1
DNA-PK	DNA-dependent protein kinase
SSBs	Single-strand breaks
DSBs	Double-strand breaks
HR	Homologous recombination
NHEJ	Non-homologous end joining
BER	Base excision repair
NER	Nucleotide excision repair
MMR	Mismatch repair
PARP	Poly(ADP-ribose) polymerase
ATRi	ATR inhibition
DC	Dendritic cell
TREX1	Three-prime repair exonuclease 1
DAMPs	Damage-associated molecular patterns
HMGB1	High mobility group box 1
ATP	Adenosine triphosphate
cGAS	Cyclic GMP-AMP synthase
STING	Stimulator of interferon genes
cGAMP	Cyclic GMP-AMP
IRF3	Interferon regulatory factor 3
NF-κB	Nuclear factor-κB
YTHDF1	YTH N6-methyladenosine RNA binding protein 1
HO-1	Heme oxygenase-1
ICIs	Immune checkpoint inhibitors
PD-1	Programmed death-1
PD-L1	Programmed death-ligand 1
CTLA-4	Cytotoxic T-lymphocyte-associated protein 4
Th1	T-helper 1
AREG	Amphiregulin
SFRT	Spatially fractionated radiotherapy
TCP	Tumour-control probability
NTCP	Normal-tissue complication probability
GARD	Genomic-adjusted radiation dose

## References

[1] Harrington K.J., Billingham L.J., Brunner T.B., Burnet N.G., Chan C.S., Hoskin P., Mackay R.I., Maughan T.S., Macdougall J., McKenna W.G. (2011). Guidelines for Preclinical and Early Phase Clinical Assessment of Novel Radiosensitisers. Br. J. Cancer.

[2] Kandemir Gursel O. (2018). Recent Technological Advances in Radiotherapy. Eur. Arch. Med. Res..

[3] Wu Y., Song Y., Wang R., Wang T. (2023). Molecular Mechanisms of Tumor Resistance to Radiotherapy. Mol. Cancer.

[4] Lomax M.E., Folkes L.K., O’Neill P. (2013). Biological Consequences of Radiation-Induced DNA Damage: Relevance to Radiotherapy. Clin. Oncol..

[5] Lynch C., Pitroda S.P., Weichselbaum R.R. (2024). Radiotherapy, Immunity, and Immune Checkpoint Inhibitors. Lancet Oncol..

[6] Ciccia A., Elledge S.J. (2010). The DNA Damage Response: Making It Safe to Play with Knives. Mol. Cell.

[7] Jackson S.P., Bartek J. (2009). The DNA-Damage Response in Human Biology and Disease. Nature.

[8] Hanahan D., Weinberg R.A. (2011). Hallmarks of Cancer: The Next Generation. Cell.

[9] Huang R.-X., Zhou P.-K. (2020). DNA Damage Response Signaling Pathways and Targets for Radiotherapy Sensitization in Cancer. Signal Transduct. Target. Ther..

[10] Wang M., Chen S., Ao D. (2021). Targeting DNA Repair Pathway in Cancer: Mechanisms and Clinical Application. MedComm.

[11] Lord C.J., Ashworth A. (2012). The DNA Damage Response and Cancer Therapy. Nature.

[12] Chen H., Han Z., Luo Q., Wang Y., Li Q., Zhou L., Zuo H. (2022). Radiotherapy Modulates Tumor Cell Fate Decisions: A Review. Radiat. Oncol..

[13] Rostami A., Lambie M., Yu C.W., Stambolic V., Waldron J.N., Bratman S.V. (2020). Senescence, Necrosis, and Apoptosis Govern Circulating Cell-Free DNA Release Kinetics. Cell Rep..

[14] Yang Y., Wu M., Cao D., Yang C., Jin J., Wu L., Hong X., Li W., Lu L., Li J. (2021). ZBP1-MLKL Necroptotic Signaling Potentiates Radiation-Induced Antitumor Immunity via Intratumoral STING Pathway Activation. Sci. Adv..

[15] Derby S.J., Chalmers A.J., Carruthers R.D. (2022). Radiotherapy-Poly(ADP-Ribose) Polymerase Inhibitor Combinations: Progress to Date. Semin. Radiat. Oncol..

[16] Bright S.J., Manandhar M., Flint D.B., Kolachina R., Kacem M.B., Martinus D.K.J., Turner B.X., Qureshi I., McFadden C.H., Marinello P.C. (2024). ATR Inhibition Radiosensitizes Cells through Augmented DNA Damage and G_2_ Cell Cycle Arrest Abrogation. JCI Insight.

[17] Kansara M., Leong H.S., Lin D.M., Popkiss S., Pang P., Garsed D.W., Walkley C.R., Cullinane C., Ellul J., Haynes N.M. (2013). Immune Response to RB1-Regulated Senescence Limits Radiation-Induced Osteosarcoma Formation. J. Clin. Investig..

[18] Jeon H.-Y., Kim J.-K., Ham S.W., Oh S.-Y., Kim J., Park J.-B., Lee J.-Y., Kim S.-C., Kim H. (2016). Irradiation Induces Glioblastoma Cell Senescence and Senescence-Associated Secretory Phenotype. Tumour Biol..

[19] Fletcher-Sananikone E., Kanji S., Tomimatsu N., Di Cristofaro L.F.M., Kollipara R.K., Saha D., Floyd J.R., Sung P., Hromas R., Burns T.C. (2021). Elimination of Radiation-Induced Senescence in the Brain Tumor Microenvironment Attenuates Glioblastoma Recurrence. Cancer Res..

[20] Saleh T., Bloukh S., Hasan M., Al Shboul S. (2023). Therapy-Induced Senescence as a Component of Tumor Biology: Evidence from Clinical Cancer. Biochim. Biophys. Acta Rev. Cancer.

[21] Tomimatsu N., Di Cristofaro L.F.M., Kanji S., Samentar L., Jordan B.R., Kittler R., Habib A.A., Espindola-Netto J.M., Tchkonia T., Kirkland J.L. (2025). Targeting cIAP2 in a Novel Senolytic Strategy Prevents Glioblastoma Recurrence after Radiotherapy. EMBO Mol. Med..

[22] Walle T., Martinez Monge R., Cerwenka A., Ajona D., Melero I., Lecanda F. (2018). Radiation Effects on Antitumor Immune Responses: Current Perspectives and Challenges. Ther. Adv. Med. Oncol..

[23] Karam S.D., Reddy K., Blatchford P.J., Waxweiler T., DeLouize A.M., Oweida A., Somerset H., Marshall C., Young C., Davies K.D. (2018). Final Report of a Phase I Trial of Olaparib with Cetuximab and Radiation for Heavy Smoker Patients with Locally Advanced Head and Neck Cancer. Clin. Cancer Res..

[24] de Haan R., van den Heuvel M.M., van Diessen J., Peulen H.M.U., van Werkhoven E., de Langen A.J., Lalezari F., Pluim D., Verwijs-Janssen M., Vens C. (2021). Phase I and Pharmacologic Study of Olaparib in Combination with High-Dose Radiotherapy with and without Concurrent Cisplatin for Non-Small Cell Lung Cancer. Clin. Cancer Res..

[25] Derby S., Jackson M.R., Williams K., Stobo J., Kelly C., Sweeting L., Shad S., Herbert C., Short S.C., Williamson A. (2024). Concurrent Olaparib and Radiation Therapy in Older Patients with Newly Diagnosed Glioblastoma: The Phase 1 Dose-Escalation PARADIGM Trial. Int. J. Radiat. Oncol. Biol. Phys..

[26] Dillon M.T., Bergerhoff K.F., Pedersen M., Whittock H., Crespo-Rodriguez E., Patin E.C., Pearson A., Smith H.G., Paget J.T.E., Patel R.R. (2019). ATR Inhibition Potentiates the Radiation-Induced Inflammatory Tumor Microenvironment. Clin. Cancer Res..

[27] Mavroeidi D., Georganta A., Panagiotou E., Syrigos K., Souliotis V.L. (2024). Targeting ATR Pathway in Solid Tumors: Evidence of Improving Therapeutic Outcomes. IJMS.

[28] Patin E.C., Nenclares P., Chan Wah Hak C., Dillon M.T., Patrikeev A., McLaughlin M., Grove L., Foo S., Soliman H., Barata J.P. (2024). Sculpting the Tumour Microenvironment by Combining Radiotherapy and ATR Inhibition for Curative-Intent Adjuvant Immunotherapy. Nat. Commun..

[29] Vendetti F.P., Pandya P., Clump D.A., Schamus-Haynes S., Tavakoli M., diMayorca M., Islam N.M., Chang J., Delgoffe G.M., Beumer J.H. (2023). The Schedule of ATR Inhibitor AZD6738 Can Potentiate or Abolish Antitumor Immune Responses to Radiotherapy. JCI Insight.

[30] Dillon M.T., Patin E.C., Mohammed K., Guevara J., Smith S.A., Dean E., Soliman H., Nenclares P., Ryugenji M., Northcote D. (2025). Tumor Control and Immune Activation through Palliative Irradiation and ATR Inhibition, PATRIOT Part C: A Phase Ib Trial. Nat. Commun..

[31] Mohiuddin I.S., Kang M.H. (2019). DNA-PK as an Emerging Therapeutic Target in Cancer. Front. Oncol..

[32] Samuels M., Falkenius J., Bar-Ad V., Dunst J., van Triest B., Yachnin J., Rodriguez-Gutierrez A., Kuipers M., You X., Sarholz B. (2024). A Phase 1 Study of the DNA-PK Inhibitor Peposertib in Combination With Radiation Therapy With or Without Cisplatin in Patients With Advanced Head and Neck Tumors. Int. J. Radiat. Oncol. Biol. Phys..

[33] van Bussel M.T.J., Awada A., de Jonge M.J.A., Mau-Sørensen M., Nielsen D., Schöffski P., Verheul H.M.W., Sarholz B., Berghoff K., El Bawab S. (2021). A First-in-Man Phase 1 Study of the DNA-Dependent Protein Kinase Inhibitor Peposertib (Formerly M3814) in Patients with Advanced Solid Tumours. Br. J. Cancer.

[34] Weber A.M., Ryan A.J. (2015). ATM and ATR as Therapeutic Targets in Cancer. Pharmacol. Ther..

[35] Al-Zoubi R.M., Garada K., Al Huneidi R., Baddar Z.M.H., Zarour A.A., Elaarag M., Al-Zoubi S.R., Al-Qudimat A.R., Al Zoubi M.S., Agouni A. (2025). ATM Inhibitors in Cancer Radiotherapy: Mechanisms, Clinical Development, and Future Directions. Eur. J. Med. Chem..

[36] Durant S.T., Zheng L., Wang Y., Chen K., Zhang L., Zhang T., Yang Z., Riches L., Trinidad A.G., Fok J.H.L. (2018). The Brain-Penetrant Clinical ATM Inhibitor AZD1390 Radiosensitizes and Improves Survival of Preclinical Brain Tumor Models. Sci. Adv..

[37] Jucaite A., Stenkrona P., Cselényi Z., De Vita S., Buil-Bruna N., Varnäs K., Savage A., Varrone A., Johnström P., Schou M. (2021). Brain Exposure of the ATM Inhibitor AZD1390 in Humans-a Positron Emission Tomography Study. Neuro-Oncology.

[38] Gorecki L., Andrs M., Korabecny J. (2021). Clinical Candidates Targeting the ATR–CHK1–WEE1 Axis in Cancer. Cancers.

[39] Yang E.S., Deutsch E., Mehmet A., Fayette J., Tao Y., Nabell L., Spencer S.A., Wang X.A., Spoljoric E.A., Zhang W. (2021). A Phase 1b Trial of Prexasertib in Combination with Chemoradiation in Patients with Locally Advanced Head and Neck Squamous Cell Carcinoma. Radiother. Oncol..

[40] Mueller S., Cooney T., Yang X., Pal S., Ermoian R., Gajjar A., Liu X., Prem K., Minard C.G., Reid J.M. (2022). Wee1 Kinase Inhibitor Adavosertib with Radiation in Newly Diagnosed Diffuse Intrinsic Pontine Glioma: A Children’s Oncology Group Phase I Consortium Study. Neurooncol. Adv..

[41] Jagsi R., Griffith K.A., Bellon J.R., Woodward W.A., Horton J.K., Ho A., Feng F.Y., Speers C., Overmoyer B., Sabel M. (2018). Concurrent Veliparib with Chest Wall and Nodal Radiotherapy in Patients with Inflammatory or Locoregionally Recurrent Breast Cancer: The TBCRC 024 Phase I Multicenter Study. J. Clin. Oncol..

[42] Sim H.-W., McDonald K.L., Lwin Z., Barnes E.H., Rosenthal M., Foote M.C., Koh E.-S., Back M., Wheeler H., Sulman E.P. (2021). A Randomized Phase II Trial of Veliparib, Radiotherapy, and Temozolomide in Patients with Unmethylated MGMT Glioblastoma: The VERTU Study. Neuro-Oncology.

[43] Kong A., Mehanna H. (2021). WEE1 Inhibitor: Clinical Development. Curr. Oncol. Rep..

[44] Chan Wah Hak C., Dillon M. (2025). Interfaces Between the DNA Damage Response and Anti-Cancer Immunity. Cancer J..

[45] Reisländer T., Groelly F.J., Tarsounas M. (2020). DNA Damage and Cancer Immunotherapy: A STING in the Tale. Mol. Cell.

[46] Vanpouille-Box C., Alard A., Aryankalayil M.J., Sarfraz Y., Diamond J.M., Schneider R.J., Inghirami G., Coleman C.N., Formenti S.C., Demaria S. (2017). DNA Exonuclease Trex1 Regulates Radiotherapy-Induced Tumour Immunogenicity. Nat. Commun..

[47] Gameiro S.R., Jammeh M.L., Wattenberg M.M., Tsang K.Y., Ferrone S., Hodge J.W. (2014). Radiation-Induced Immunogenic Modulation of Tumor Enhances Antigen Processing and Calreticulin Exposure, Resulting in Enhanced T-Cell Killing. Oncotarget.

[48] Zhu M., Yang M., Zhang J., Yin Y., Fan X., Zhang Y., Qin S., Zhang H., Yu F. (2021). Immunogenic Cell Death Induction by Ionizing Radiation. Front. Immunol..

[49] Gupta A., Probst H.C., Vuong V., Landshammer A., Muth S., Yagita H., Schwendener R., Pruschy M., Knuth A., van den Broek M. (2012). Radiotherapy Promotes Tumor-Specific Effector CD8+ T Cells via Dendritic Cell Activation. J. Immunol..

[50] Colangelo N.W., Gerber N.K., Vatner R.E., Cooper B.T. (2024). Harnessing the cGAS-STING Pathway to Potentiate Radiation Therapy: Current Approaches and Future Directions. Front. Pharmacol..

[51] Li J., Yang H., Zhu M., Zhang P., Liu Y., Niu Y., Zhou T., Li Y. (2025). Unlocking the Therapeutic Potential of the STING Signaling Pathway in Anti-Tumor Treatment. Clin. Exp. Med..

[52] Pan J., Fei C.-J., Hu Y., Wu X.-Y., Nie L., Chen J. (2023). Current Understanding of the cGAS-STING Signaling Pathway: Structure, Regulatory Mechanisms, and Related Diseases. Zool. Res..

[53] Deng L., Liang H., Burnette B., Beckett M., Darga T., Weichselbaum R.R., Fu Y.-X. (2014). Irradiation and Anti–PD-L1 Treatment Synergistically Promote Antitumor Immunity in Mice. J. Clin. Investig..

[54] Bakhoum S.F., Ngo B., Laughney A.M., Cavallo J.-A., Murphy C.J., Ly P., Shah P., Sriram R.K., Watkins T.B.K., Taunk N.K. (2018). Chromosomal Instability Drives Metastasis through a Cytosolic DNA Response. Nature.

[55] Li J., Hubisz M.J., Earlie E.M., Duran M.A., Hong C., Varela A.A., Lettera E., Deyell M., Tavora B., Havel J.J. (2023). Non-Cell-Autonomous Cancer Progression from Chromosomal Instability. Nature.

[56] Benci J.L., Xu B., Qiu Y., Wu T.J., Dada H., Victor C.T.-S., Cucolo L., Lee D.S.M., Pauken K.E., Huang A.C. (2016). Tumor Interferon Signaling Regulates a Multigenic Resistance Program to Immune Checkpoint Blockade. Cell.

[57] Brown M.C., Low J.T., Bowie M.L., Ashley D.M. (2024). Taking the STING out of Radiotherapy: STING Checkpoints Mediate Radiation Resistance. J. Clin. Investig..

[58] Wen C., Wang L., Piffkó A., Chen D., Yu X., Zawieracz K., Bugno J., Yang K., Naccasha E.Z., Ji F. (2024). YTHDF1 Loss in Dendritic Cells Potentiates Radiation-Induced Antitumor Immunity via STING-Dependent Type I IFN Production. J. Clin. Investig..

[59] Zhang C., Deng Z., Wu J., Ding C., Li Z., Xu Z., Chen W., Yang K., Wei H., He T. (2024). HO-1 Impairs the Efficacy of Radiotherapy by Redistributing cGAS and STING in Tumors. J. Clin. Investig..

[60] Grapin M., Richard C., Limagne E., Boidot R., Morgand V., Bertaut A., Derangere V., Laurent P.-A., Thibaudin M., Fumet J.D. (2019). Optimized Fractionated Radiotherapy with Anti-PD-L1 and Anti-TIGIT: A Promising New Combination. J. Immunother. Cancer.

[61] Demaria S., Guha C., Schoenfeld J., Morris Z., Monjazeb A., Sikora A., Crittenden M., Shiao S., Khleif S., Gupta S. (2021). Radiation Dose and Fraction in Immunotherapy: One-Size Regimen Does Not Fit All Settings, so How Does One Choose?. J. Immunother. Cancer.

[62] Young K.H., Baird J.R., Savage T., Cottam B., Friedman D., Bambina S., Messenheimer D.J., Fox B., Newell P., Bahjat K.S. (2016). Optimizing Timing of Immunotherapy Improves Control of Tumors by Hypofractionated Radiation Therapy. PLoS ONE.

[63] Melia E., Parsons J.L. (2023). DNA Damage and Repair Dependencies of Ionising Radiation Modalities. Biosci. Rep..

[64] Oike T., Niimi A., Okonogi N., Murata K., Matsumura A., Noda S.-E., Kobayashi D., Iwanaga M., Tsuchida K., Kanai T. (2016). Visualization of Complex DNA Double-Strand Breaks in a Tumor Treated with Carbon Ion Radiotherapy. Sci. Rep..

[65] Burckel H., Nicol A., Mura C., Rousseau M., Bou-Gharios J., Froidurot L., Richard C., Morgand V., Laurent P.-A., Limagne E. (2025). Distinct Immune Responses to Proton and Photon Radiotherapy: Implications for Anti-PD-L1 Combination Therapy in Colorectal Cancer. J. Transl. Med..

[66] Fernández Muñoz M., Bishr M.K., O’Leary B., Melcher A., Harrington K. (2025). Modulating T-Cell Responses to Enhance the Effects of Radiotherapy. Cancer J..

[67] Talebi F., Gregucci F., Ahmed J., Ben Chetrit N., Brown B.D., Chan T.A., Chand D., Constanzo J., Demaria S., Gabrilovich D.I. (2025). Updates on Radiotherapy-Immunotherapy Combinations: Proceedings of 8th Annual ImmunoRad Conference. OncoImmunology.

[68] Postow M.A., Callahan M.K., Barker C.A., Yamada Y., Yuan J., Kitano S., Mu Z., Rasalan T., Adamow M., Ritter E. (2012). Immunologic Correlates of the Abscopal Effect in a Patient with Melanoma. N. Engl. J. Med..

[69] Zhang X., Chen Y., Liu X., Li G., Zhang S., Zhang Q., Cui Z., Qin M., Simon H.-U., Terzić J. (2025). STING in Cancer Immunoediting: Modeling Tumor-Immune Dynamics throughout Cancer Development. Cancer Lett..

[70] Gray L.H., Conger A.D., Ebert M., Hornsey S., Scott O.C. (1953). The Concentration of Oxygen Dissolved in Tissues at the Time of Irradiation as a Factor in Radiotherapy. Br. J. Radiol..

[71] Brand A., Singer K., Koehl G.E., Kolitzus M., Schoenhammer G., Thiel A., Matos C., Bruss C., Klobuch S., Peter K. (2016). LDHA-Associated Lactic Acid Production Blunts Tumor Immunosurveillance by T and NK Cells. Cell. Metab..

[72] Chang C.-H., Qiu J., O’Sullivan D., Buck M.D., Noguchi T., Curtis J.D., Chen Q., Gindin M., Gubin M.M., van der Windt G.J.W. (2015). Metabolic Competition in the Tumor Microenvironment Is a Driver of Cancer Progression. Cell.

[73] Riaz N., Alban T.J., Haddad R.I., Saul M., Makarov V., Zhu Y., Cohen E.E.W., Ferris R.L., Chang P.M.-H., Lin J.-C. (2026). Tumor Ecosystem and Microbiome Features Associated with Efficacy and Resistance to Avelumab plus Chemoradiotherapy in Head and Neck Cancer. Nat. Cancer.

[74] Mukherjee D., Romano E., Walshaw R., Zeef L.A.H., Banyard A., Kitcatt S.J., Cheadle E.J., Tuomela K., Pendharkar S., Al-Deka A. (2023). Reprogramming the Immunosuppressive Tumor Microenvironment Results in Successful Clearance of Tumors Resistant to Radiation Therapy and Anti-PD-1/PD-L1. OncoImmunology.

[75] Piffkó A., Pitroda S.P., Liang H.L., Weichselbaum R.R. (2025). Amphiregulin as a Mediator of Radiation-Induced Systemic Effects in Metastatic Cancer: Balancing Local Control with Distant Progression. Clin. Cancer Res..

[76] Darragh L.B., Karam S.D. (2026). Radiation as an Immune Modulator: Mechanisms and Implications for Combination with Immunotherapy. Nat. Rev. Cancer.

[77] Wilk J., Box C., Harrington K., Nill S., Oelfke U. (2025). Alternative Radiotherapy Delivery Approaches to Modulate Radiation Response. Cancer J..

[78] Bekker R.A., Obertopp N., Redler G., Penagaricano J., Caudell J.J., Yamoah K., Pilon-Thomas S., Moros E.G., Enderling H. (2024). Spatially Fractionated GRID Radiation Potentiates Immune-Mediated Tumor Control. Radiat. Oncol..

[79] Jiang L., Li X., Zhang J., Li W., Dong F., Chen C., Lin Q., Zhang C., Zheng F., Yan W. (2021). Combined High-Dose LATTICE Radiation Therapy and Immune Checkpoint Blockade for Advanced Bulky Tumors: The Concept and a Case Report. Front. Oncol..

[80] Bergeron P., Milliat F., Deutsch E., Mondini M. (2024). Heterogeneous Intratumor Irradiation: A New Partner for Immunotherapy. OncoImmunology.

[81] Bergeron P., Dos Santos M., Sitterle L., Tarlet G., Lavigne J., Liu W., Gerbé de Thoré M., Clémenson C., Meziani L., Schott C. (2024). Non-Homogenous Intratumor Ionizing Radiation Doses Synergize with PD1 and CXCR2 Blockade. Nat. Commun..

[82] McMillan M.T., Khan A.J., Powell S.N., Humm J., Deasy J.O., Haimovitz-Friedman A. (2024). Spatially Fractionated Radiotherapy in the Era of Immunotherapy. Semin. Radiat. Oncol..

[83] Chen J., Levy A., Tian A.-L., Huang X., Cai G., Fidelle M., Rauber C., Ly P., Pizzato E., Sitterle L. (2025). Low-Dose Irradiation of the Gut Improves the Efficacy of PD-L1 Blockade in Metastatic Cancer Patients. Cancer Cell.

[84] Luo J., Zhi Q., Li D., Xu Y., Zhu H., Zhao L., Ren G., Wang J., Liu N. (2025). Low Dose Radiotherapy Combined with Immune Checkpoint Inhibitors Induces Ferroptosis in Lung Cancer via the Nrf2/HO-1/GPX4 Axis. Front. Immunol..

[85] Dong J., Qi Y., Sha S. (2025). Low-Dose Radiation Improves Tumor Immune Microenvironment, Enhancing the Effects of Anti-CTLA-4 Therapy. Iran. J. Immunol..

[86] Laurent P.-A., Shi L., Bouarroudj L., Benzazon N., Gerbé De Thoré M., Liu W., Aglave M., Bergeron P., Naulin F., Sitterlé L. (2025). Low-Dose Radiotherapy Enhances the Efficacy of PD-L1 Blockade and Induces the Abscopal Effect. J. Immunother. Cancer.

[87] Verginadis I.I., Citrin D.E., Ky B., Feigenberg S.J., Georgakilas A.G., Hill-Kayser C.E., Koumenis C., Maity A., Bradley J.D., Lin A. (2025). Radiotherapy Toxicities: Mechanisms, Management, and Future Directions. Lancet.

[88] Gkikoudi A., Triantopoulou S., Markellou E., Xynou V., Vasilopoulos S.N., Myronakis M., Laiakis E.C., Theodorou K., Terzoudi G.I., Georgakilas A.G. (2026). From Cellular Radiosensitivity to Precision Radiotherapy: Integrating Functional Assays, Genomics, and Clinical Modeling. Cancers.

[89] Mohile N.A., Messersmith H., Gatson N.T., Hottinger A.F., Lassman A., Morton J., Ney D., Nghiemphu P.L., Olar A., Olson J. (2022). Therapy for Diffuse Astrocytic and Oligodendroglial Tumors in Adults: ASCO-SNO Guideline. J. Clin. Oncol..

[90] Perry J.R., Laperriere N., O’Callaghan C.J., Brandes A.A., Menten J., Phillips C., Fay M., Nishikawa R., Cairncross J.G., Roa W. (2017). Short-Course Radiation plus Temozolomide in Elderly Patients with Glioblastoma. N. Engl. J. Med..

[91] Yom S.S., Torres-Saavedra P., Caudell J.J., Waldron J.N., Gillison M.L., Xia P., Truong M.T., Kong C., Jordan R., Subramaniam R.M. (2021). Reduced-Dose Radiation Therapy for HPV-Associated Oropharyngeal Carcinoma (NRG Oncology HN002). J. Clin. Oncol..

[92] Rubini D., Gagliardi F., Menditti V.S., D’Ambrosio L., Gallo P., D’Onofrio I., Pisani A.R., Sardaro A., Rubini G., Cappabianca S. (2024). Genetic Profiling in Radiotherapy: A Comprehensive Review. Front. Oncol..

[93] Bodalal Z., Trebeschi S., Nguyen-Kim T.D.L., Schats W., Beets-Tan R. (2019). Radiogenomics: Bridging Imaging and Genomics. Abdom. Radiol..

[94] Zhang B., Chang K., Ramkissoon S., Tanguturi S., Bi W.L., Reardon D.A., Ligon K.L., Alexander B.M., Wen P.Y., Huang R.Y. (2017). Multimodal MRI Features Predict Isocitrate Dehydrogenase Genotype in High-Grade Gliomas. Neuro. Oncol..

[95] Chang K., Bai H.X., Zhou H., Su C., Bi W.L., Agbodza E., Kavouridis V.K., Senders J.T., Boaro A., Beers A. (2018). Residual Convolutional Neural Network for the Determination of IDH Status in Low- and High-Grade Gliomas from MR Imaging. Clin. Cancer Res..

[96] Rosenstein B.S. (2017). Radiogenomics: Identification of Genomic Predictors for Radiation Toxicity. Semin. Radiat. Oncol..

[97] Seibold P., Behrens S., Schmezer P., Helmbold I., Barnett G., Coles C., Yarnold J., Talbot C.J., Imai T., Azria D. (2015). XRCC1 Polymorphism Associated with Late Toxicity After Radiation Therapy in Breast Cancer Patients. Int. J. Radiat. Oncol. Biol. Phys..

[98] Young A., O’Leary B. (2026). Molecular Biology of Radiation Response and Resistance. Curr. Opin. Oncol..

[99] Scott J.G., Sedor G., Ellsworth P., Scarborough J.A., Ahmed K.A., Oliver D.E., Eschrich S.A., Kattan M.W., Torres-Roca J.F. (2021). Pan-Cancer Prediction of Radiotherapy Benefit Using Genomic-Adjusted Radiation Dose (GARD): A Cohort-Based Pooled Analysis. Lancet Oncol..

[100] Chiang C.L., Chan K.S.K., Li H., Ng W.T., Chow J.C.H., Choi H.C.W., Lam K.O., Lee V.H.F., Ngan R.K.C., Lee A.W.M. (2024). Using the Genomic Adjusted Radiation Dose (GARD) to Personalize the Radiation Dose in Nasopharyngeal Cancer. Radiother. Oncol..

[101] Xia H., Li Z., Lin Y., Lin Y., Zeng L., Xu B., Yao Q., Zheng R. (2024). Validation of a Genome-Based Model for Adjusting Radiotherapy Dose (GARD) in Patients with Locally Advanced Rectal Cancer. Sci. Rep..

[102] Grimes D.R. (2022). Limitations of the Radiosensitivity Index as a Direct Prognostic Marker. Lancet Oncol..

[103] Bratman S.V., Milosevic M.F., Liu F.-F., Haibe-Kains B. (2017). Genomic Biomarkers for Precision Radiation Medicine. Lancet Oncol..

[104] Wekking D., Silva C.A.C., Viscò R., Denaro N., Lambertini M., Maccioni A., Loddo E., Willard-Gallo K., Scartozzi M., Derosa L. (2025). The Interplay between Gut Microbiota, Antibiotics, and Immune Checkpoint Inhibitors in Patients with Cancer: A Narrative Review with Biological and Clinical Aspects. Crit. Rev. Oncol. Hematol..

[105] Routy B., Le Chatelier E., Derosa L., Duong C.P.M., Alou M.T., Daillère R., Fluckiger A., Messaoudene M., Rauber C., Roberti M.P. (2018). Gut Microbiome Influences Efficacy of PD-1-Based Immunotherapy against Epithelial Tumors. Science.

[106] Wang M., Rousseau B., Qiu K., Huang G., Zhang Y., Su H., Le Bihan-Benjamin C., Khati I., Artz O., Foote M.B. (2024). Killing Tumor-Associated Bacteria with a Liposomal Antibiotic Generates Neoantigens That Induce Anti-Tumor Immune Responses. Nat. Biotechnol..

